# Combining cues to judge distance and direction in an immersive virtual reality environment

**DOI:** 10.1167/jov.21.4.10

**Published:** 2021-04-26

**Authors:** Peter Scarfe, Andrew Glennerster

**Affiliations:** 1 University of Reading, Earley Gate, Whiteknights Road, Reading, Berkshire, UK; 2 University of Reading, Earley Gate, Reading, UK

**Keywords:** direction, distance, virtual reality, cue combination, Bayesian

## Abstract

When we move, the visual direction of objects in the environment can change substantially. Compared with our understanding of depth perception, the problem the visual system faces in computing this change is relatively poorly understood. Here, we tested the extent to which participants’ judgments of visual direction could be predicted by standard cue combination rules. Participants were tested in virtual reality using a head-mounted display. In a simulated room, they judged the position of an object at one location, before walking to another location in the room and judging, in a second interval, whether an object was at the expected visual direction of the first. By manipulating the scale of the room across intervals, which was subjectively invisible to observers, we put two classes of cue into conflict, one that depends only on visual information and one that uses proprioceptive information to scale any reconstruction of the scene. We find that the sensitivity to changes in one class of cue while keeping the other constant provides a good prediction of performance when both cues vary, consistent with the standard cue combination framework. Nevertheless, by comparing judgments of visual direction with those of distance, we show that judgments of visual direction and distance are mutually inconsistent. We discuss why there is no need for any contradiction between these two conclusions.

## Introduction

### Three-dimensional representation in a moving observer

The coordinates of three-dimensional (3D) vision can seem misleadingly simple. Two coordinates define the visual direction of a point as viewed, for example, by the cyclopean eye, the third defines the distance of the point along that line of sight. Most research in the field of 3D vision focuses on the cues that contribute to the estimation of distance and depth presumably because, for a static observer, such as is typical for most psychophysical experiments, the estimation of visual direction seems simpler and less to do with the representation of the 3D world around us. However, for a moving observer in a static world, or a static observer viewing moving objects, the situation is quite different (Wexler et al., 2001). Objects change both their depth and their visual direction and, in both cases, if an observer is to perceive a stable 3D world, they must take account of changes in the visual direction of objects just as much as they do for changes in object depth.

There is good evidence that people are able to update their estimate of the visual direction of previously viewed objects when they move to a new location (Foo et al., 2005; Klatzky et al., 2003; Klier et al., 2008; Loomis et al., 1998; Medendorp, 2011; Rieser & Rider, 1991; Siegle et al., 2009; Thompson et al., 2004). To do this accurately requires two things: first, an estimate of the translation of the observer, which may come from a range of cues in addition to vision, including audition, proprioception, and somatosensory information, all of which must be integrated together (Mayne, 1974; Siegle et al., 2009); second, it requires an ability to use this information appropriately to update the observer's representation of the scene and the observer's location in it (whatever form that representation might take). Loomis et al. describe this as updating a “spatial image” (Giudice et al., 2011; Loomis et al., 2007).

Here, we focus on the role of two quite distinct potential signals about visual direction that might contribute to the spatial updating process. One is purely visual and the other involves information about the distance between the optic center of the eye/camera in two or more locations. First, we describe what we call physical-based cues. In the field of photogrammetry (Hartley & Zisserman, 2000; Shapiro et al., 1995), in which the 3D layout of a static scene is recovered from a series of images as a camera moves through the scene, the 3D structure of the scene can be reconstructed but only up to scale. In other words, the metric or Euclidean structure of the scene can be recovered from the images alone, but it is not possible to make any comment about the overall scale of the reconstruction until information is provided about the distance between two or more optic centers along the path of the camera/eye. Such information could come from proprioception; for example, information from the leg muscles indicating that the observer had walked a meter or, equivalently, information about the interocular separation that, in combination with vergence information, indicates fixation distance (Backus & Matza-Brown, 2003; Brenner & van Damme, 1998; Howard, 2008; Mon-Williams et al., 2000; Richards & Miller, 1969; Swenson, 1932; Tresilian & Mon-Williams, 2000; Tresilian et al., 1999).

It is this scaling information that is missing from a purely visual, photogrammetric reconstruction. If people build a scaled reconstruction of the scene, including the location of a target that they have to track, and if we assume that they have access to proprioceptive information about how far they have walked then, in theory, a participant with this information could close their eyes and continually judge the direction of the target as they moved in the environment (Frissen et al., 2011; Loomis et al., 1992; Loomis & Philbeck, 2008; Siegle et al., 2009). We describe this strategy as using physical-based cues, because it is entirely based on a scaled reconstruction of both the scene and of the participant's location in that reconstruction. These two factors together determine the physical-based direction of the target. Although many separate factors may contribute to this estimate, we treat them as all contributing to a single cue. This practice is consistent with the standard definition of a cue as “any sensory information that gives rise to a sensory estimate” (Ernst & Bulthoff, 2004, p. 163).

The second set of cues we consider (and, again, we group these together and consider them as a single entity), are what we call texture-based cues. At the opposite extreme from physical-based cues, we can assume that people use only the images that arrive that the eye to reconstruct their environment, as in unscaled photogrammetry. In this case, if the entire scene is doubled in size about the cyclopean point (so everything gets larger and further away) and if there is no information available to the system about the length of the baseline (that is, distance between the camera/eye locations, e.g. interocular separation), then there is no way for the system to detect that the scene has changed size. Participants can still navigate; they can still tell where they are in the unscaled photogrammetric reconstruction, and they can still judge the visual direction of remembered objects in that reconstruction. We describe this estimate as texture-based because, when the room expands, so do all the textures (the bricks on the wall, the tiles on the floor) and a person's judgment of the target visual direction is entirely based on these, not the physical size of the room.

The distinction between scaled and unscaled reconstruction of a scene has some similarities to the distinction between absolute and relative disparities, although it is not identical. The absolute disparity of a point is the binocular disparity between it and the location where the two eyes are fixated, whereas the relative disparity between two points is the difference between their absolute disparities and so is independent of the vergence angle of the eyes (Harris, 2004; Howard & Rogers, 2002). However, doubling interocular separation (or, equivalently in this experiment, shrinking the scene by a factor of two around the cyclopean point) doubles both the absolute disparities *and* the relative disparities of points but it only affects physical-based cues, not texture-based cues, so the absolute/relative distinction does not map onto the two cues we have described. The distinction between scaled and unscaled reconstruction is important in photogrammetry and it clarifies the information that is available to the visuomotor system under different assumptions.

When judging visual direction, if the scene expands or contracts about the cyclopean point between 1) the moment when the participant initially sees the target and 2) the moment when they have to make a judgment about the visual direction of the previously viewed target (after having walked to a new location), then physical-based and texture-based cues will conflict, but crucially they both provide estimates in the same units (visual angle) and so can be combined.

Previously, we have investigated the effect of these two types of cue to distance using an immersive virtual reality environment where the scene can expand or contract around the observer's cyclopean point. In those experiments, we have demonstrated how these cues could be combined according to the widely accepted rules of cue combination (Glennerster et al., 2006; Rauschecker et al., 2006; Svarverud et al., 2012; Svarverud et al., 2010). However, in these instances we have always examined them in relation to the perception of depth. In the current work, we extend this analysis to the perception of visual direction and, to anticipate our results, we show again that performance in combined cue situations for visual direction follows the pattern expected by standard cue combination rules, just as it did for perceived depth.

Here, we also compare judgments of direction and distance from the two classes of cue, because there are clear ways in which both types of judgment could adhere to standard cue combination rules yet produce perceptual estimates that are mutually inconsistent. First, observers might differentially weight the available cues when making each type of judgment. Second, judgments of direction and distance might be differentially biased. By bias, we are referring to a discrepancy between 1) the value of a property of the external world (real or simulated) and 2) the person's estimate of that property. Elsewhere, this concept has been termed external accuracy (Burge et al., 2010). A similar conclusion about perceptual inconsistencies has been advocated on the basis of evidence showing that the integration of sensory cues does not necessarily lead to the calibration of those same cues (Smeets et al., 2006).

### Aims of the current study

The current study had two aims. First, we examine whether judgments of visual direction in freely moving observers could be predicted using a simple weighted averaging of the two types of cue we have described (physical-based and texture-based), as has been shown for judgments of distance (Svarverud et al., 2010). We adopt the most widely accepted framework for considering sensory cue combination (weighted averaging) for this purpose (Landy et al., 1995; Maloney & Landy, 1989). This posits that the goal of combining sensory cues is to maximize the precision of the combined cues estimate and construct a single unified internal representation of the scene in units such as depth (Landy et al., 1995). Second, we explore the possibility that judgments of distance and direction could be mutually inconsistent. Using immersive virtual reality allows us to examine these aims in a naturalistic spatial updating task, while parametrically controlling the cues available to the observer (Scarfe & Glennerster, 2015, 2019). Spatial updating has generally been discussed in terms of a geometric representations of space and an observer's location in that space rather than the weighted combination of conflicting estimates of visual direction (e.g., Klatzky et al., 1998), although see Nardini et al. (2008) for a similar approach to weighting visual-only or proprioceptive-based cues but applied to a homing task.

## Methods

### Participants

Nine observers took part in experiments, including one author (P.S.). The experiments were reviewed and approved by the University of Reading Research Ethics Committee. Observers were paid £10 per hour to take part. Participants ranged in age from 18 to 33. All had normal or corrected-to-normal vision (Snellen acuity of 6/6 or better).

### General methods

Observers viewed the virtual scene binocularly using a NVIS SX111 head-mounted display (HMD). This had a vertical field of view of 72°, horizontal field of view of 102°, and 50° of horizontal binocular overlap. The displays for each eye had a resolution of 1,280 × 1,024 pixels and a refresh rate of 60 Hz. The position and orientation of the HMD was tracked using a 14 camera Vicon tracking system (MX3 and T20S cameras). The HMD was calibrated such that the left and right eyes viewing frustums could be calculated from the six degrees of freedom tracking data. This allowed geometrically correct perspective projection of the 3D scene as the observer moved through the virtual environment (Gilson et al., 2011). The HMD was driven by two computers connected directly by gigabit Ethernet, one for running the tracking software and one for generating the simulated environment. The tracking computer had a quad core Intel Xeon 3.6GHz CPU, NVidia Quadro K2000 graphics and 8 GB of RAM. This ran Vicon Tracker 2.0.1 software that outputted the coordinates of the HMD at 240 Hz. The graphics computer had an eight core AMD Opteron 6212 CPU, dual NVidia GeForce GTX 590 Graphics cards and 16 GB RAM. This polled the coordinates of the HMD at 60 Hz using the Vicon DataStream SDK for Matlab (R2013b).

Stimuli were rendered online in OpenGL using Matlab and the Psychophysics toolbox extensions (Brainard, 1997; Kleiner et al., 2007; Pelli, 1997). The dual displays of the HMD were driven from a single graphics card port using a Matrox multidisplay adaptor (“TripleHead2Go”) to ensure the two eyes images were updated synchronously, without tearing. This created a single virtual 2,560 × 1,024 screen (left half mirrored to the left eyes HMD display, and the right half the right mirrored to the eyes HMD display). Observer responses were recorded with a handheld wireless button box connected to the graphics computer via Bluetooth.

### Visual direction: Stimulus and task

**Figure 1.**
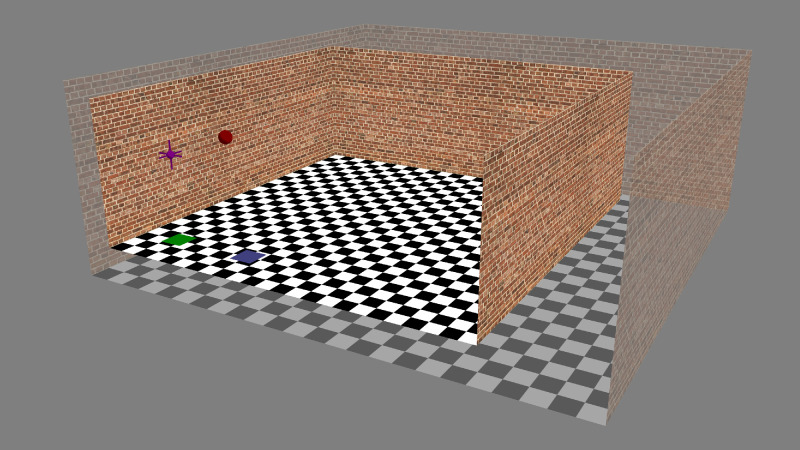
Rendering of the virtual room, with the back wall removed. The green square indicates the position of view zone 1 (where the observer stood in interval 1) and the blue square the position of view zone 2 (where the observer stood in interval 2), in the “near wall” condition (colored squares were not present in the simulated VR environment). During the experiment observers judged the distance and visual direction of a red sphere rendered in front of view zone 1. The purple fiducial marker above view zone 1 represents the cyclopean position of the observer. During the experiment the room could dynamically expand or contact around the observers’ cyclopean point (semi-opaque room shows an example of expansion). When the room expanded the bricks on the wall and tiles on the floor scaled in size such that from the cyclopean point the room looked identical before and after it changed in size. This allowed “texture-based” and “physical-based” cues to conflict with one another (see main text for details). Supplementary Movie 1 shows a dynamic version of this figure.

The experiment took place in a virtual room (Figure 1) with brick textured walls and a black and white checkerboard textured floor (24 × 24 tiles). The checkerboard was created procedurally in Matlab and the brick texture was loaded in from a photo. The photo was tiled, resized, and filtered in Adobe Photoshop CS6 to create a single image with no visible seams between the edges of the tiled image. Both images were converted to OpenGL textures for rendering.

**Figure 2.**
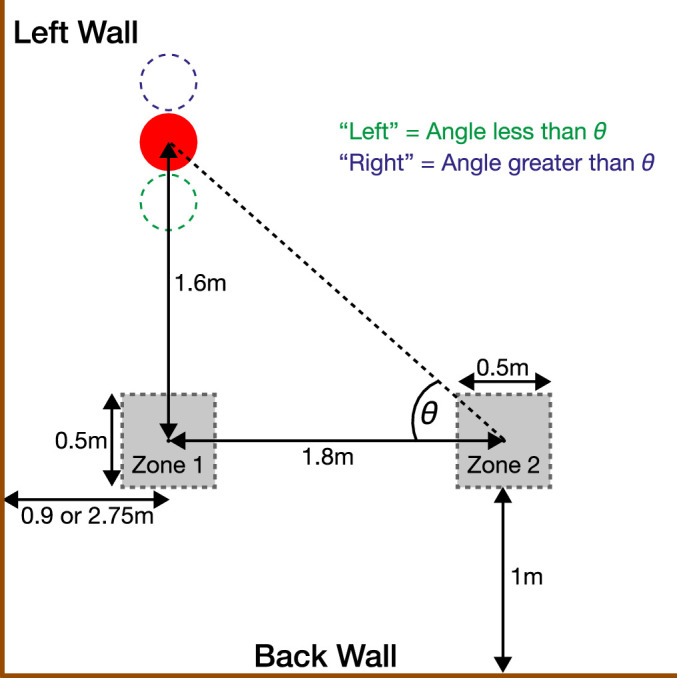
Schematic view of a portion of the room from above. The position of the view zones and red sphere are shown relative to the left and back walls of the room. In interval 1, observers remembered the position of the sphere from view zone 1. They then walked across the room to view zone 2, where they saw a second sphere that could be in the same physical location or translated along the line joining the center of zone 1 and the center of the sphere in interval 1 (dashed circles). The task was to judge whether the visual direction of the second sphere was to the “left” or “right” of the original sphere.

Each trial began with the observer in a simulated room with rendered physical dimensions of 8 m × 8 m × 3 m (width, depth, and height). The floor of the rendered room was concordant with the physical floor of the laboratory. Interval 1 was initiated when the participants entered an invisible 50-cm square view zone on the left side of the simulated room (view zone 1). View zone 1 was always 1 m from the back wall but could be either 0.9 m or 2.75 m from the left-hand side simulated wall (Figure 2). We label these conditions “near wall” and “near middle,” respectively. These two conditions were used because previous research has shown that, in an environment such as this, proximity to the wall modulates the weighting observers assign to visual cues such as disparity, motion, and texture (Svarverud et al., 2012).

Upon entering view zone 1 a red target sphere (30 cm in diameter) appeared at 1.6 m directly in front of the view zone. The height of the sphere was set to that of the observer's cyclopean eye, which was recorded at the moment they entered the view zone. The coordinates of the cyclopean eye were computed directly from the left and right eye OpenGL model view matrices on each refresh cycle (60 Hz). Observers were informed that the ball would always be directly in front of view zone 1 before starting the experiment. The sphere was matt, red in color, and lit by a white light source providing ambient and diffuse illumination positioned 2 m above and 2 m to its right. The rest of the room was unaffected by this light source.

The sphere was viewable continuously, so long as the *X* and *Z* coordinates of the observer's cyclopean point remained in the view zone. If the observer moved out of the view zone, the ball disappeared, but it reappeared as soon as they reentered. Observers were instructed to get a good idea of the position of the ball and were encouraged to move relative to the ball to aid this process, for example, by rocking side to side laterally. Once they were confident of the position of the ball, they pressed a button on the button box. This action triggered the HMD screens to go blank for 0.5 seconds, after which time the rendered room reappeared, without the red sphere. When the room reappeared, it could have a different size (either smaller or larger) to that in the first interval. When the room changed size between intervals, it uniformly scaled in size around the cyclopean point of the observer (Figure 1, Supplementary Movie 1).

As a result, the checkerboard and brick textures scaled with the room so that there were the same number of bricks on the walls and tiles on the floor when the room changed size. The textures were magnified using bilinear sampling and minified using a tri-linear sampling (multiresolution mipmap pyramid), both with 16× anisotropic filtering. This ensured that there was no visual texture aliasing or excessive blur at different scales, even when viewing the textures at very shallow viewing angles. In addition to this, the whole scene was anti-aliased using full screen multisampling with eight samples per image pixel. Thus, the cyclopean viewpoint when the room reappeared was identical to that at the end of the first interval, except for any small movement the observer made during the short blank period. When a room is scaled in this way, people remain consciously unaware that the room has changed size, even though this size change can be dramatic (Glennerster et al., 2006; Svarverud et al., 2010).

Once the room reappeared, observers walked rightwards across the room 1.8 m to view zone 2 (also 1 m from the back wall). A physical bar 1 m in height, not rendered in the virtual environment, straddled the diameter of the laboratory at right angles to the walls and provided a guide for walking. The positions of both view zones were marked on the bar with physical markers so that observers could feel when to stop walking by touch. The distance walked was always 1.8 m and observers rapidly became accustomed to this movement distance. This also meant that on different trials observers walked across different proportions of the simulated room. All observers were debriefed at the end of the experiment and, consistent with previous research (Glennerster et al., 2006), none reported being aware that the room scaled in size, or that they were traversing different proportions of the simulated room. At best, the only thing observers noticed was that on some trials they felt as if they were moving faster or slower than others.

When observers entered view zone 2, a new ball appeared in the room (interval 2). This ball was also directly in front of view zone 1; however, its distance from view zone 1 (in the *Z* direction) was generally different from that in interval 1 (the full psychophysical procedure for this is described further elsewhere in this article). The observers’ task was to judge whether, in terms of visual direction, the second ball was to the left or the right of the first ball. In Figure 2, this corresponds to whether θ was smaller or greater than that in interval 1. Before the beginning of the experiment, the task was explained clearly to observers with the aid of diagrams and each observer completed practice blocks until they were confident in the task. With a short period of practice, observers became very proficient in the task, finding it quick and intuitive to perform. We consider that a task in which the target disappears and then participants judge the location of a probe relative to the remembered location is the cleanest way to measure their representation of perceived location. When the target can be seen continuously, biases are introduced relative to participants’ perception in a static scene (Tcheang et al., 2005; Wallach et al., 1974).

The size of the ball in interval 2 was scaled relative to the room so that observers would be unable to identify the physical scale of the room simply by judging the size of the ball relative to the room across intervals. In addition to this, the ball's size was jittered by a random value drawn from a uniform distribution of ±15%. This prevented participants from using the angular size of the ball as an accurate proxy for viewing distance, although we note that this does not completely eliminate angular size as a potential cue to viewing distance. Observers were informed that the ball's size would vary randomly across trials, but regardless of the ball's size, the task was always to judge its visual direction. If participants had misattributed some of the change in ball size to a change in viewing distance (e.g., 50%), this would be equivalent to carrying out the experiment at a different viewing distance. Assuming, as we do throughout, that participants remember this viewing distance from interval 1, and use it in their estimation of viewing distance or direction in interval 2, then there is only a very small effect of this potential misattribution on the quantities we compute later on. Specifically, the maximum possible effect will be for trials that have the largest jitter. For these extreme trials, the estimated ratio of reliabilities for texture-based and physical-based cues would change by 5% from the ratio that would have been computed otherwise. Overall, the mean effect across all trials would be zero and the standard deviation of the change in ratio would be 0.2%.

Upon recording their response with a button press, the rendering of the room was extinguished. This cued the observer to walk back to view zone 1. When they got half-way between view zones 1 and 2, they reentered the simulated room of interval 1 for the next trial of the experiment.

### Visual direction: Single cue sensitivities

To test the weighted averaging model, we first measured single cue sensitivities to changes in visual direction. We did this using a paradigm in which one cue, either physical-based or texture-based was varied, and the other one was constant. We did this both when the participant was near the wall and near the middle of the room. As described elsewhere in this article, physical-based cues refer to information from stereo and motion parallax that is scaled by an estimate of the baseline between views (such as interocular separation or distance walked), which can potentially signal the position of the sphere independent of other aspects of the room. Texture-based cues, in contrast, signal the position of the ball relative to other aspects of the room, for example, the tiled floor and brick walls. Disparity and motion parallax can also contribute to these, for example, the ratio of two relative disparities is independent of any estimate of the interocular separation. Single cue sensitivities were measured by holding one cue constant while letting the other vary when the room scaled in size across intervals 1 and 2. Elsewhere in this article, we detail how we can infer single cue sensitivities from measurements with both cues present. For both classes of cue, in interval 1 the ball was always positioned 1.6m directly in front of view zone 1. However, in interval 2 the positioning of the ball differed.

When measuring a physical threshold, the position of the ball in front of zone 1 remained the same relative to the room (i.e., a different physical position compared with interval 1). In contrast, when measuring a texture threshold, the ball remain at the same physical position in front of zone 1 (i.e., a different position relative to the room compared with interval 1). By varying the magnitude of room scaling between intervals 1 and 2, we were able to map out a psychometric function for each type of cue, for each position in the room: 1) texture cues near wall, 2) physical cues near wall, 3) texture cues near middle, and 4) physical cues near middle. Whereas in previous research observers were instructed to pay explicit attention to the type of cue being varied (Svarverud et al., 2010), in the present study we gave no instructions in this regard, so as to measure observers’ natural propensity to use each type of cue in each condition.

There are clearly many different components that contribute to the overall sensitivity to a cue, including the variability in measuring the distance walked, variability in remembering the distance of the target in interval 1, and variability in the process of computing an angle from these two distances. This is true for a large range of studies in this area (see Hillis et al., 2004 for a discussion). When we fit a cumulative Gaussian to the psychometric function for one of these tasks, we are assuming that a number of sources of noise sum together (variances add, for independent noise sources) to give a total noise for all the components contributing to performance. For both physical-based and texture-based cues, variability in measuring the viewing distance, measuring the distance walked, or computing an angle from these distances are all likely sources of noise. The key aim is to see, under these common assumptions, how well weighted averaging can account for the data.

For each of the four functions, the scale factor of the room in the second interval was determined by the psi-marginal adaptive method (Prins, 2013), implemented in the Palamedes toolbox for Matlab (Prins & Kingdom, 2009). The psi-marginal method is based on the psi method (Kontsevich & Tyler, 1999), but allows the experimenter to label the four parameters of the psychometric function as parameters of interest or nuisance parameters. The psi-marginal method marginalizes over the nuisance parameters so that stimulus placement only gathers information about these if they provide information about the parameters of interest (for a full description, see Prins, 2013). Because we were primarily interested in the point of subjective equality (PSE) and slope of the psychometric function these were set as parameters of interest, whereas the lapse and guess rate were set as nuisance parameters. As such, the PSE and slope of the psychometric function were set as our free parameters when psychometric functions were fit (as discussed elsewhere in this article).

The minimum and maximum room scale factors from which the psi-marginal could be selected were set at 0.35 and 3.95. Collecting data at values high and low on the psychometric function is key for gaining well-fit, unbiased, functions (Prins, 2013; Wichmann & Hill, 2001a, 2001b). We chose these values because they had resulted in near asymptotic behavior and well fit functions during piloting. Trials for each for the four psychometric functions were randomly interleaved and completed in blocks of 40 (10 trials per function per block). There were 5 blocks, giving a total of 150 trials per psychometric function. Observers took breaks between blocks, but typically completed two blocks back to back. The experiment could be completed over multiple days or weeks, depending on the availability of the observer.

### Visual direction: Measuring perceived visual direction when both cues vary

Next, we measured perceived visual direction when both classes of cue could vary between intervals 1 and 2. If the weighted averaging model holds, we should be able to predict cue weighting in the combined-cue case from single cue sensitivities. For this part of the experiment we used five scale factors with equal logarithmic spacing (2^−1^, 2^−0.5^, 2^0^, 2^0.5^, and 2^1^), corresponding with scale factors of 0.5, 0.71, 1.0, 1.41, and 2.0. The values were chosen to lie well within the scale factors used for measuring single cue sensitivities, so as to not be extrapolating beyond the range in which cue weights were measured. For each scale factor, we collected a separate psychometric function using the psi-marginal adaptive method. Here, the psi-marginal method was used to vary the depth of the sphere in interval 2. This had the effect of altering the visual angle θ for both cues concurrently (Figure 2). All five of these psychometric functions were run randomly interleaved within blocks. Each block consisted of 50 trials, 10 per function. There were in total 15 experimental blocks, giving a total of 150 trials per psychometric function, that is, the same number of trials per function as when measuring single cue sensitivities. All other aspects of the task were identical to that described elsewhere in this article.

### Perceived distance: Stimulus and task

The aim of the second part of the experiment was to obtain estimates of the perceived distance of the target sphere in the same experimental conditions with as with judgments of visual direction. Five observers also made distance judgments (in subsequent figures: PS, S1, S3, S4, and S5) based on their availability to take part. The stimulus and methodology were largely identical to judgments of visual direction, so we just describe the differences here. In the first interval, observers judged the distance to the red sphere in front of them (distance being the *Z* dimension shown Figure 2). Once they were confident, they pressed a button that triggered the HMD screens to go blank for 0.5 seconds, after which the rendered room reappeared, with a new red sphere. The observer's task was to judge whether the red sphere in the second interval was closer or further from them in the Z dimension compared with the red sphere in interval 1.

As with the direction task, the room could scale in size around the cyclopean point between intervals and the red ball scaled in size relative to the room with an additional jitter of value drawn from a uniform distribution ±15%. In addition to this, the ball in the second interval was jittered laterally in the *X* dimension by a value drawn from a uniform distribution ±25.43 cm. This was equivalent to ±9° at the 1.6 m distance of the ball in interval 1. This lateral jitter was introduced so as to disrupt the use of monocular cues to complete the task. Observers were informed of this random lateral jitter and told that, regardless of the jitter value, the task was always to judge distance in the *Z* dimension.

As with the judgments of direction, there were two parts. In the first part, we measured single cue sensitivities for distance by holding target distance (as defined by one cue) constant while the distance of the target as defined by the other cue was varied. As before, the room scale factor between intervals 1 and 2 was determined adaptively by the psi-marginal method. In the second part, we measured perceived distance with the same five room expansion factors. Here the psi-marginal method was used to measure the distance of the ball from view zone 1 in the *Z* dimension. Observers were given practice trials until they were familiar with the task and confident in carrying it out before beginning the experiment. All other aspects of the procedure were identical to the direction task.

### Definition of cues for perceived visual direction

In measuring single cue sensitivities for physical and texture cues to visual direction, we parametrically varied the scale of the room between intervals 1 and 2, as has been done previously for judgments of distance (Svarverud et al., 2010). However, room scale factor has a nonlinear relationship with the property that the observer was asked to estimate, namely, whether the visual direction of the ball had changed between intervals 1 and 2. For single cue sensitivities to be correctly measured there must be a linear relationship between 1) the property being estimated and 2) the property being manipulated. There is currently active debate surrounding the cues observers use to complete a given task and whether or not they are linearly related to the judgment that an observer is making (Hillis et al., 2004; Rosas et al., 2004; Saunders & Chen, 2015; Todd, 2015; Todd et al., 2010). Often, this remains an open question (Rosas et al., 2004). With this aim in mind, before fitting psychometric functions to the data, we converted the room scale factor into a change in visual direction for each class of cue. In the following, we describe this process and in doing so the predictions that would follow if an observer relied entirely on either class of cue.

In measuring a threshold for physical cues, when the room scale between intervals 1 and 2, the ball remained in the same position relative to the room. As a result, the ball's physical position changed between intervals. Here, we describe what that would look like to an observer who relied 100% on physical cues. In interval 1, the observer estimates the distance to the ball *D* from view zone 1. In interval 2, they walk across the room by distance *B* to view zone 2. The room has scaled in size by a factor of *S_i_* between intervals 1 and 2 (where *S_i_* varies between trials and 0.5 < *S_i_* < 2), but because the observer relies 100% on physical cues, they ignore this scaling and expect that the visual direction of the ball in view zone 2 will be:

(3) $$\theta_{1}^{Phy}=\tan^{-1}\left(\frac{D}{B}\right)$$

Thus, even though the observer in interval 1 is at zone 1, with the target in front of them, Equation 3 refers to the expectation of the angle θ to the target sphere as viewed from zone 2, but under the stimulus conditions present in interval 1. However, in interval 2, room scaling causes the ball's physical *distance* from view zone 1 to change from *D* to D*S_i_. Thus, when the ball reappears, its angle from physical cues is given by

(4) $$\theta_{2}^{Phy}=\tan^{-1}\left(\frac{D*S_{i}}{B}\right)$$

The difference in angle between intervals 1 and 2 for an observer who relied 100% on physical cues would therefore be:

(5) $$\begin{array}{ccc} \Delta \theta^{Phy} & = & \theta_{2}^{Phy}-\theta_{1}^{Phy}=\tan^{-1}\left(\frac{D*S_{i}}{B}\right)\\ & & -\;\tan^{-1}\left(\frac{D}{B}\right) \end{array}$$

Note that, if an observer relied 100% on texture cues, we would be unable to measure a threshold for physical cues because each trial would look identical to the observer.

In measuring a threshold for texture cues, when the room scales between intervals 1 and 2, the ball remains in the same physical position. As a result, the ball's position relative to the room changes between intervals. Here we describe what this would look like to an observer who relied 100% on texture cues. In interval 1, the observer estimates the distance to the ball (*D*) relative to a distance that is defined in terms of the room (*R*), which can refer to any property of the room, for example, the size of one of the square tiles on the floor or the distance from the observer to the back wall since these all covary. Thus, *D*/*R* is unitless and gives a measure of the distance of the target that remains independent of the overall scaling of the room. In interval 2, the observer walks across the room by distance *B* to view zone 2, again judging this distance relative to the same property of the room. The observer's expectation of the ball's visual direction at view zone 2 is therefore given by:

(6) $$\theta_{1}^{Tex}=\tan^{-1}\left(\frac{D/R}{B/R\;}\right)=\tan^{-1}\left(\frac{D}{B\;}\right)$$

Because the ball remains in the same physical position when the room scales, its distance relative to view zone 1 changes inversely to the rooms scale (*D*/*R*)/*S_i_*.

(7) $$\;\theta_{2}^{Tex}=\tan^{-1}\left(\frac{\left(D/R\right)*\frac{1}{S_{i}}}{B/R\;}\right)=\tan^{-1}\left(\frac{D}{B*S_{i}}\right)$$

The difference in angle between intervals 1 and 2 for an observer who relied 100% on texture cues would therefore be:

(8) $$\begin{array}{ccc} \Delta \theta^{Tex} & = & \theta_{2}^{Tex}-\theta_{1}^{Tex}=\tan^{-1}\left(\frac{D}{B*S_{i}}\right)\\ & & -\;\tan^{-1}\left(\frac{D}{B\;}\right) \end{array}$$

Note that, if an observer relied 100% on physical cues, we would be unable to measure a threshold for texture cues because each trial would look identical to the observer (*D*  and *B* are both unchanged across trials). It is also important to note that $\theta_{2}^{Tex}$ refers to the angle at which the observer would see the ball from zone 2, if zone 2 had been scaled with the room (hence *B*/*R* on the denominator is not multiplied by $\frac{1}{S_{i}}$ in the same way that *D* is, so that *D* remains fixed in physical coordinates). It does not matter that the visual direction judgment takes place from a physically different place (namely, zone 2 at distance *B* from zone 1). The idea is that, as the observer walks from zone 1 to zone 2 with a constant place in mind where they think that the ball was in interval 1, there should be some measure to describe that constant location, even though its visual direction changes as the observer walks. We have chosen, for the sake of convenience, the visual direction of that location as seen from zone 2, if zone 2 had been scaled with the room, hence Equation 7.

### Definition of cues for perceived distance

In measuring a threshold for physical cues for distance, when the room scale changed between intervals 1 and 2, the ball remained in the same position relative to the room. As a result, the ball's physical position changed between intervals. Here we describe what that would look like to an observer who relied 100% on physical cues. In interval 1, the observer estimates the distance *D* to the ball from view zone 1. In interval 2 they again estimate distance, but in a room that has scaled in by a factor of *S_i_*. Because the observer relies 100% on physical cues, they ignore this scaling. Thus, their estimate of distance in interval 1 is assumed to be:

(9) $$D_{1}^{Phy}=D$$

and in interval 2 room scaling causes the ball's physical distance from view zone 1 to change from *D* to D*S_i_:

(10) $$D_{2}^{Phy}=D*S_{i}$$

So, the difference in estimated distance of the target between intervals 1 and 2 for an observer who relied 100% on physical cues would therefore be:

(11) $$\Delta D^{Phy}=D_{2}^{Phy}-D_{1}^{Phy}=D\left(1-\;S_{i}\right)\;$$

These equations are similar to Equations 3 through 5 earlier, but they refer only to the distance part (the left-hand edge of the triangle shown in Figure 2), rather than θ. Just as in the case of direction judgments, if an observer relied 100% on texture cues, they would be unable to detect any change between intervals 1 and 2 whatever the value of *S_i_* and hence we could not measure a threshold in this experiment.

As for direction thresholds, measuring a distance threshold for texture cues requires that, when the room scales between intervals 1 and 2, the ball remains in the same physical position. Consequently, the ball's position relative to the room changes between intervals. Here we describe what this would look like to an observer who relied 100% on texture cues. In interval 1, the observer estimates the distance to the ball (*D*). In interval 2, the observer's judgment of distance is given by:

(12) $$D_{1}^{Tex}=D$$

This is similar to Equation 6, which applied to direction but here only refers to distance (the left-hand edge of the triangle shown in Figure 2). In interval 2, the target distance relative to view zone 1 changes inversely to the room's scale (while its physical distance remains constant):

(13) $$D_{2}^{Tex}=\frac{D}{S_{i}}$$

Hence, for an observer who relied only on texture cues, the magnitude of the distance signal (the difference between intervals 1 and 2) would be:

(14) $$\Delta D^{Tex}=D_{2}^{Tex}-D_{1}^{Tex}=D\left(1-\frac{1}{S_{i}}\right)\;$$

## Results

### Perceived direction: Single cues

After conversion, cumulative Gaussian functions were fitted to observers’ data by maximum likelihood in Matlab using the Palamedes software package with the mean and slope of the function as free parameters. These parameters correspond to the parameters of interest set in the psi-marginal adaptive procedure (Prins & Kingdom, 2009). The point of subjective equality (mean of the fitted cumulative Gaussian) and slope of the fitted function were estimated with 95% confidence intervals (CIs) computed via parametric bootstrapping (1,000 bootstrap samples). The standard deviation of the cumulative Gaussian is given by the inverse of the slope. The standard deviation of the fitted cumulative Gaussian is useful for determining the sensitivity of observers to that cue. We explain how we use this value to obtain an estimate of the reliability of the cue elsewhere in this article (Equations 17–20).

**Figure 3.**
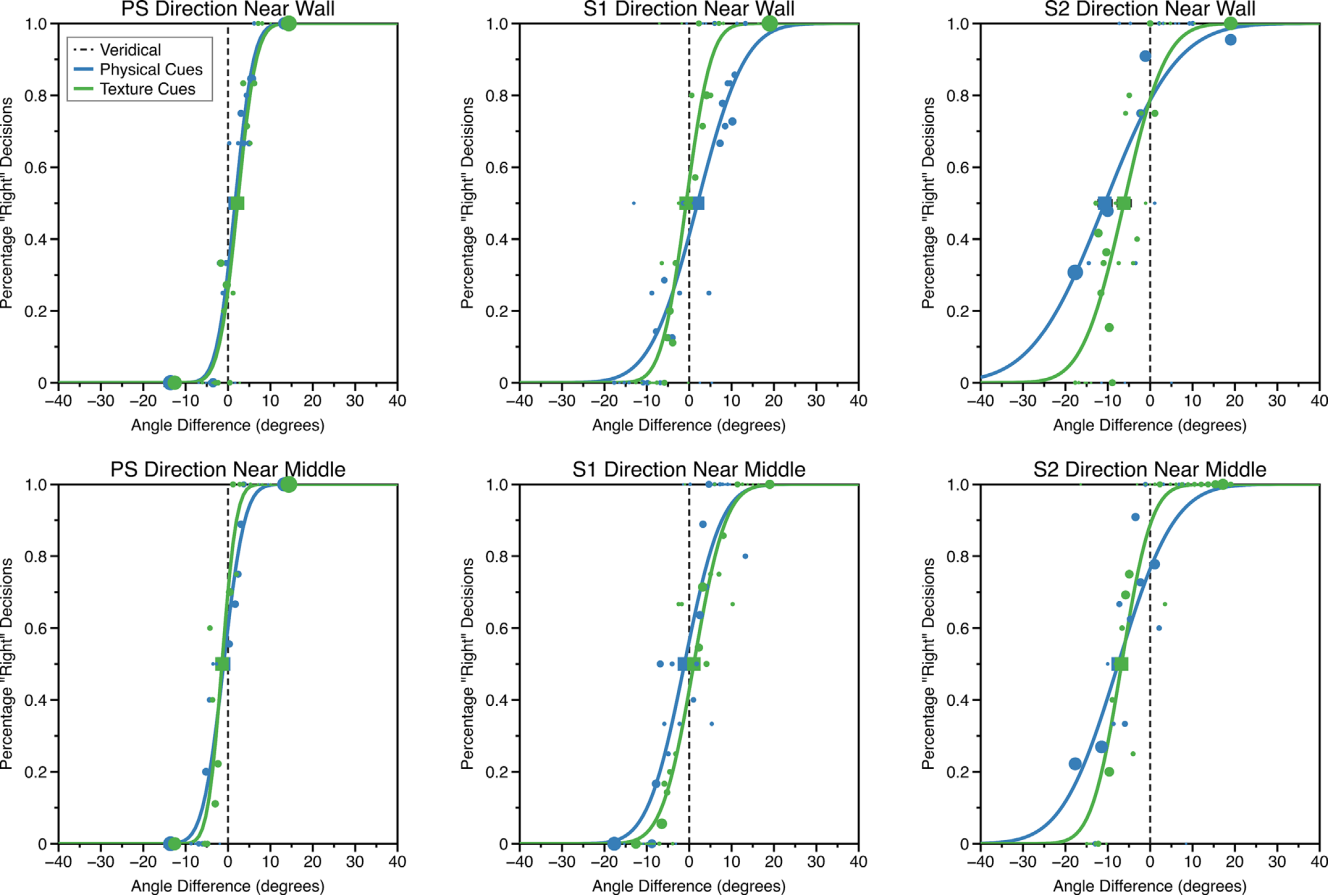
Example psychometric functions for physical and texture cues for judgments of visual direction (upper row “near wall” condition, lower row “near middle” condition). The area of the circular markers is proportional to the number of trials at that stimulus level, as determined by the psi-marginal adaptive procedure. The squares represent the point of subjective equality (mean of the psychometric function). Error bars around the PSE show bootstrapped 95% confidence intervals (where these are not visible, they are smaller than the size of the marker). The vertical black dashed line shows “veridical” performance; the PSEs of the psychometric functions would fall on this line if the observer was unbiased in judging visual direction.

Psychometric functions for the three representative observers are shown in Figure 3. The observers shown in Figure 3 have different levels of experience. PS is one of the authors, S1 is an experienced psychophysical observer naïve to the purposes of the experiment, and S2 a naïve observer with little to no experience of psychophysical experiments.

Clearly, it is only possible to fit functions to observers’ data if they give some weighting to the cue that is being varied. If not, observers’ responses will not be related to changes in that cue's value, making it impossible to fit a function. There were three observers where we were unable to fit a function to one or more conditions due to them performing at chance across the whole stimulus range. For observer S3, we were unable to fit a function to measure physical cue thresholds for either position in the room (near wall or near middle), for S4 we were unable to fit a physical cue function for the near middle condition, and for S5 we were unable to fit a texture cue function for the near middle condition. Therefore, in subsequent calculations, we set cue weight to zero in these conditions for these participants.

Potentially, we could have altered the scale of the room over a wider range to map out a psychometric function, but we chose not to do this for two reasons. First, we could not shrink the room any further without the physical position of view zone 2 lying beyond the right-hand side wall of the scaled room. This would have resulted in subjects walking through the wall and out of the room if it shrunk further. Second, the maximum scale factor we were using already produces a room with over double the floor area of a basketball court; thus, it seemed unlikely that these observers would be able give weight to the cues with further increases. A more plausible explanation was that observers were simply not giving any weight to the visual information contributing to that particular threshold.

### Visual direction: When both cues vary

**Figure 4.**
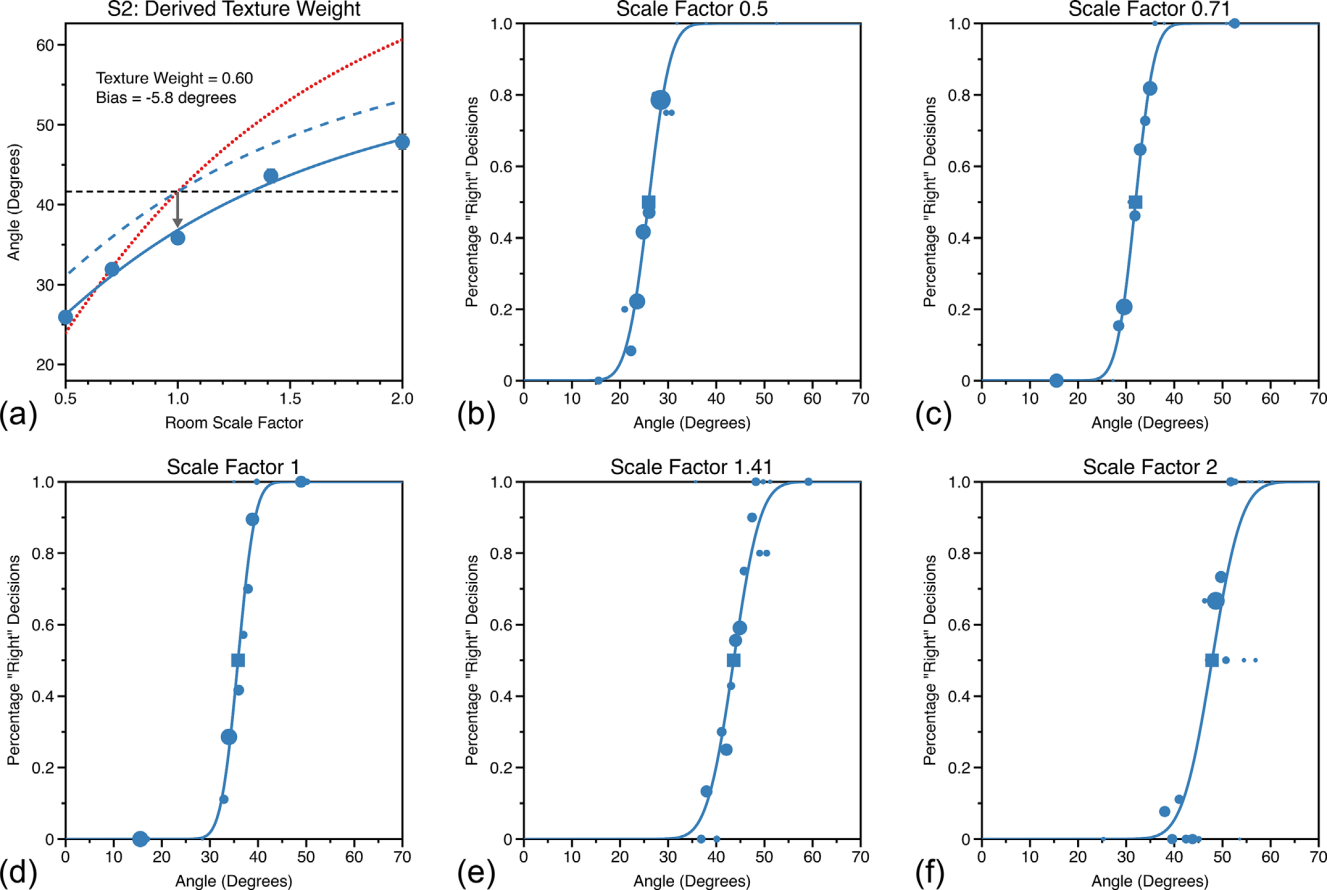
Example psychometric functions for judgments of visual direction while varying both classes of cue and for each of the five room scale factors (b–f). In each of these plots, the area of the circular data points is proportional to the number of trials presented at the that stimulus level (as determined by the psi-marginal adaptive procedure). Function PSEs are shown as squares, with error-bars showing bootstrapped 95% confidence intervals (where these cannot be seen they are smaller than the symbol size). In (a) these PSEs are plotted against room scale factor along with a fit of Equation 15 (blue dots and line), which was used to determine relative cue weighting (k) and level of perceptual bias (inset text). The horizontal dashed black line shows the prediction if an observer gave zero weight to texture-based cues, the dotted red line shows the prediction if the observer gave zero weight to physical cues (where both predictions assume no bias in perceptual estimates). The dashed blue line shows the predicted perceived visual angle if the observer weighted the cues as they did in the experiment but exhibited no perceptual bias (bias shown as dark grey arrow).

Psychometric functions for perceived visual direction for each of the five room scale factors were fitted using the same procedure as described above. Example psychometric functions for each scale factor, for observer S2 in the near wall condition are shown in Figure 4b–f. To estimate the weight given to texture when making visual direction judgments we fitted the PSEs, from the different room scale factors with a linear model (Equation 15).

(15) A=kT+c

Here, *T* is the texture cue prediction of visual direction so both *A* and *T* are vectors with five elements for the five scale factors we used. The multiplicative free parameter *k* represents the texture weight, whereas *c* is an additive parameter of the linear model incorporating any bias. The solid line in Figure 4a shows an example fit for observer S1 for the near wall condition. This observer preferentially weights texture when making judgments of visual direction. The dotted red line shows the predicted visual direction if the observer gave full weight to texture cues while the black dashed line shows predicted visual direction if the observer gave full weight to physical cues. The physical cue prediction is simply the physically correct angle, $\theta_{}^{Phy}=41.6^{\circ }$, whereas the texture cue prediction is given by

(16) $$\theta_{i}^{Tex}=\tan^{-1}\left(\frac{B}{D/S_{i}}\right)$$

Here, $\theta_{i}^{Tex}$ is the texture cue prediction of θ and varies with room scale factor *S_i_*, *B* is physical distance to the target ball, and *D* the physical distance from view zone 1 to 2. The dashed blue line in Figure 4a shows the predicted performance if the observer weighted cues by the same amount to that found, but exhibited no perceptual bias.

**Figure 5.**
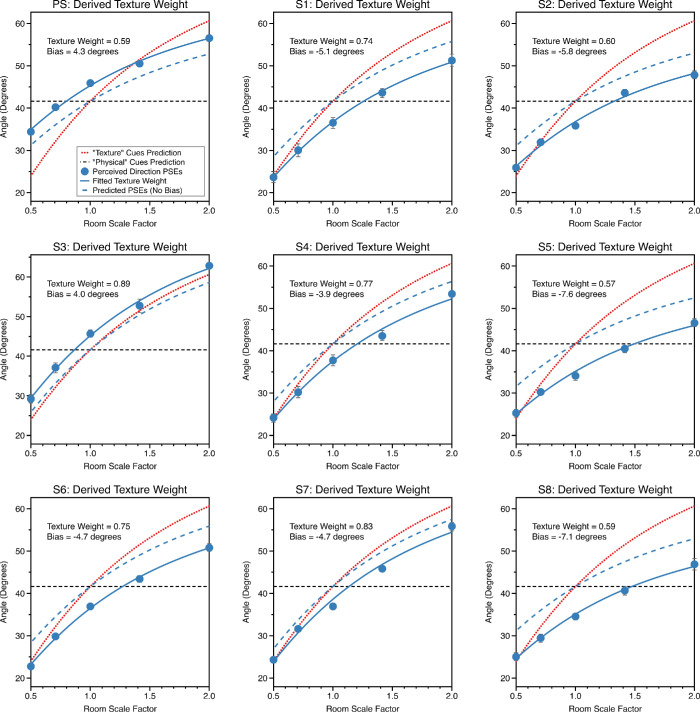
PSEs are plotted against room scale factor for the “near wall” condition when both cues were varied, for each of the nine observers. Plots are formatted as described for Figure 4a.

**Figure 6.**
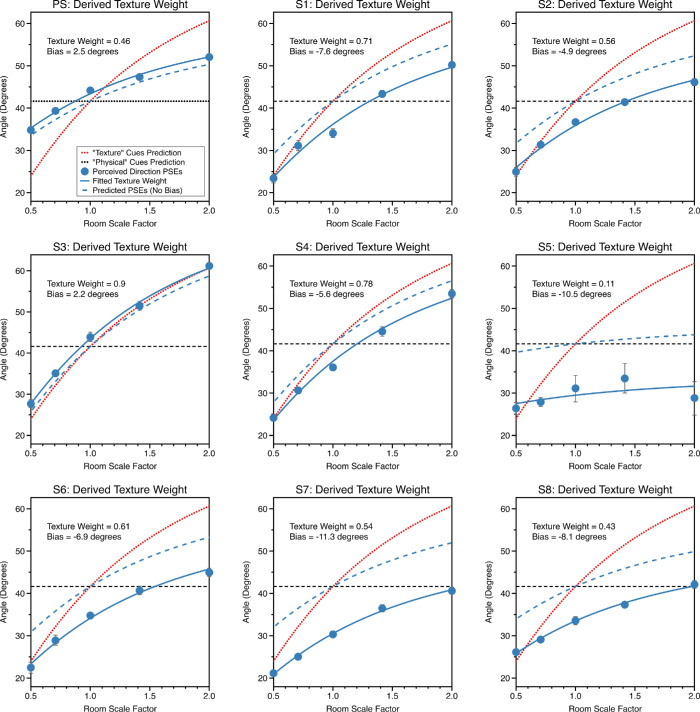
PSEs are plotted against room scale factor for the “near middle” condition when both cues were varied, for each of the nine observers. Plots are formatted as described for Figure 4a.

Comparable plots for all observers are shown in Figures 5 and 6 for the near wall and near middle conditions. Observers tended to weight texture-based cues more highly in both room positions. The average weighting for texture-based cues was 0.71 near wall and 0.57 in the near middle. This differed significantly from equal cue weighting in the near wall condition, *t* = 5.1, df = 8, 95% CI = 0.61–0.80, *p* = 9.32 × 10^−4^, but not in the “near middle” condition, t = 0.9, df = 8, 95% CI = 0.39–0.74, *p* = 0.4. In the section “Visual direction: Do observers optimally combine texture-based and physical-based cues?”, we examine whether the relative reliabilities of texture-based and physical-based cues can predict these responses.

**Figure 7.**
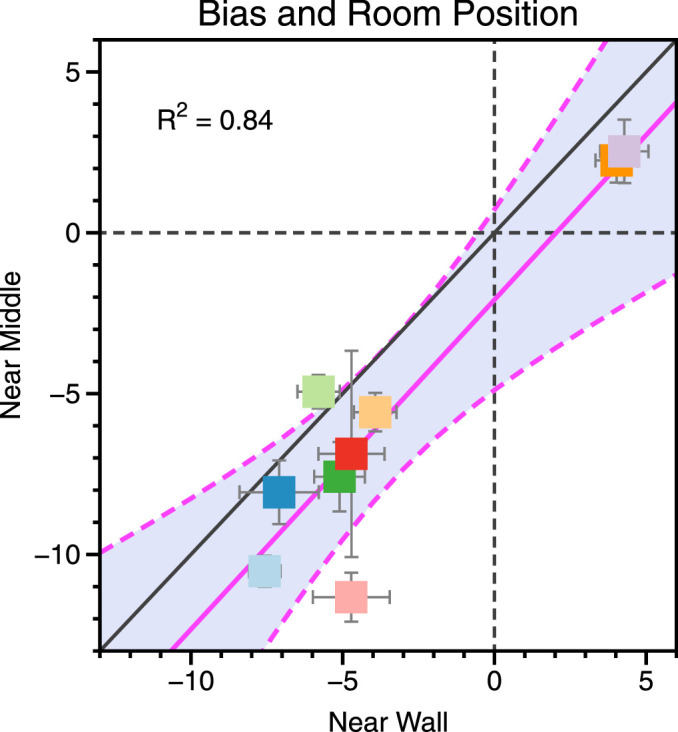
The bias in judgments of visual direction observed for the “near wall” and “near middle” conditions, when both cues varied (as in Figures 5 and 6). If observers exhibited no perceptual bias, all data would fall at the intersection of the vertical and horizontal dashed lines. The pink line shows a linear fit to the data with associated 95% confidence bounds (R^2^ value of the fit shown in text inset). Negative values in the lower left quadrant indicate an underestimation of visual direction.

Overall, when visual direction from both cues varied observers tended to underestimate visual angle. Seven of nine observers underestimated visual direction in both room positions, whereas two observers overestimated visual direction in both room positions. The average bias was −3.40° near wall and −5.57° near middle. This bias was significantly different from zero both in the near wall condition, t = −2.30, df = 8, 95% CI = −6.81 to 0.01, *p* = 0.05, and near middle conditions, t = −3.37, df = 8, 95% CI = −9.37 to −1.75, *p* = 0.01. There was a significant linear relationship between the bias observers exhibited in each room position, F_(1, 7)_ = 36.69, *p* < 0.001 (Figure 7).

### Visual direction: Do observers optimally combine texture-based and physical-based cues?

Given the assumptions of the weighted averaging model, it should be possible to predict the weighting of texture-based and physical-based cues based on the single cue sensitivities we have measured (Equation 10). However, we cannot simply use the standard deviation of the cumulative Gaussian measured in the single cue sensitivity experiments (Figure 3) as an estimate of the reliability of each cue, 1/σ^2^ . This is because both cues were present during these measurements of sensitivity, one signaling zero change from intervals 1 to 2, the other providing a signal that changed across intervals and allowed the participant to carry out the task. Assuming cue combination occurs, the zero-change cue will still affect performance as, on every trial, it will decrease the perceived perturbation of the target relative to the perturbation of the signal or changing cue that the experimenter has added (i.e., the values plotted on the *x*-axis of the psychometric functions in Figure 3). The extent of this reduction in the effective magnitude of the signal cue is determined as follows.

First, we can see how the weights of the two cues affects the measured standard deviation of the cumulative Gaussian in the comparison task. Specifically, for any given trial, ${\hat{D}}_{A}$ is the magnitude of the changing or signal cue in the experiment, ${\hat{D}}_{B}$ is the magnitude of the static cue (zero in this case) and ${\hat{D}}_{C}$ is the combined perceptual estimate (Landy et al., 1995). *w_A_* is the weight given to the changing signal cue and *w_B_* the weight given to the static cue. Thus, the presence of ${\hat{D}}_{B}$ will have the effect of pulling ${\hat{D}}_{C}$ toward zero on every trial, always by the same proportion.

(17) $${\hat{D}}_{C}=w_{A}{\hat{D}}_{A}+w_{B}{\hat{D}}_{B}$$

As a result, the experimentally measured standard deviation, ${\hat{\sigma }}_{A}$, is overestimated relative to the true underlying standard deviation σ_*A*_. It is overestimated rather than underestimated because a higher value of ${\hat{D}}_{A}$ needs to be presented than would otherwise be the case if the static cue ${\hat{D}}_{B}$ were not presented to give rise to the same internal response (i.e., reach the same value on a fitted cumulative Gaussian curve). The magnitude of this overestimation is $\frac{1}{w_{A}}$. A derivation of this is given in Scarfe (2020, pp. 49–53). So, if each individual point on a psychometric function is shifted toward the mean (PSE) by the same proportion (e.g., 50%) then the standard deviation of the cumulative Gaussian fit through the shifted data will reduce (by 50% in this example) but the mean (PSE) will not change. As a consequence, the measured standard deviation ${\hat{\sigma }}_{A}$ is given by:

(18) $${\hat{\sigma }}_{A}\;=\;\frac{\sigma_{A}}{w_{A}}$$

Since

(19) $$\frac{w_{A}}{w_{B}}=\frac{\sigma_{B}^{2}}{\sigma_{A}^{2}}$$

the ratio of observed standard deviations for the two cues from the sensitivity experiments is given by

(20) $$\frac{{\hat{\sigma }}_{A}}{{\hat{\sigma }}_{B}}=\frac{\sigma_{A}w_{B}}{\sigma_{B}w_{A}}=\frac{\sigma_{A}^{3}}{\sigma_{B}^{3}}$$

This means that the underlying ratio of reliabilities, $\frac{\sigma_{A}^{2}}{\sigma_{B}^{2}},\;$will be closer to unity than the ratio measured using the standard deviations of the cumulative Gaussians shown in Figure 3, $\frac{{\hat{\sigma }}_{A}^{2}}{{\hat{\sigma }}_{B}^{2}}$. A number of papers which have measured cue sensitivities whilst holding a conflicting cue constant would need to apply this correction to accurately estimate cue reliabilities (Svarverud, et al. 2012; Murphy et al., 2013). For an extended discussion see Scarfe (2020).

**Figure 8.**
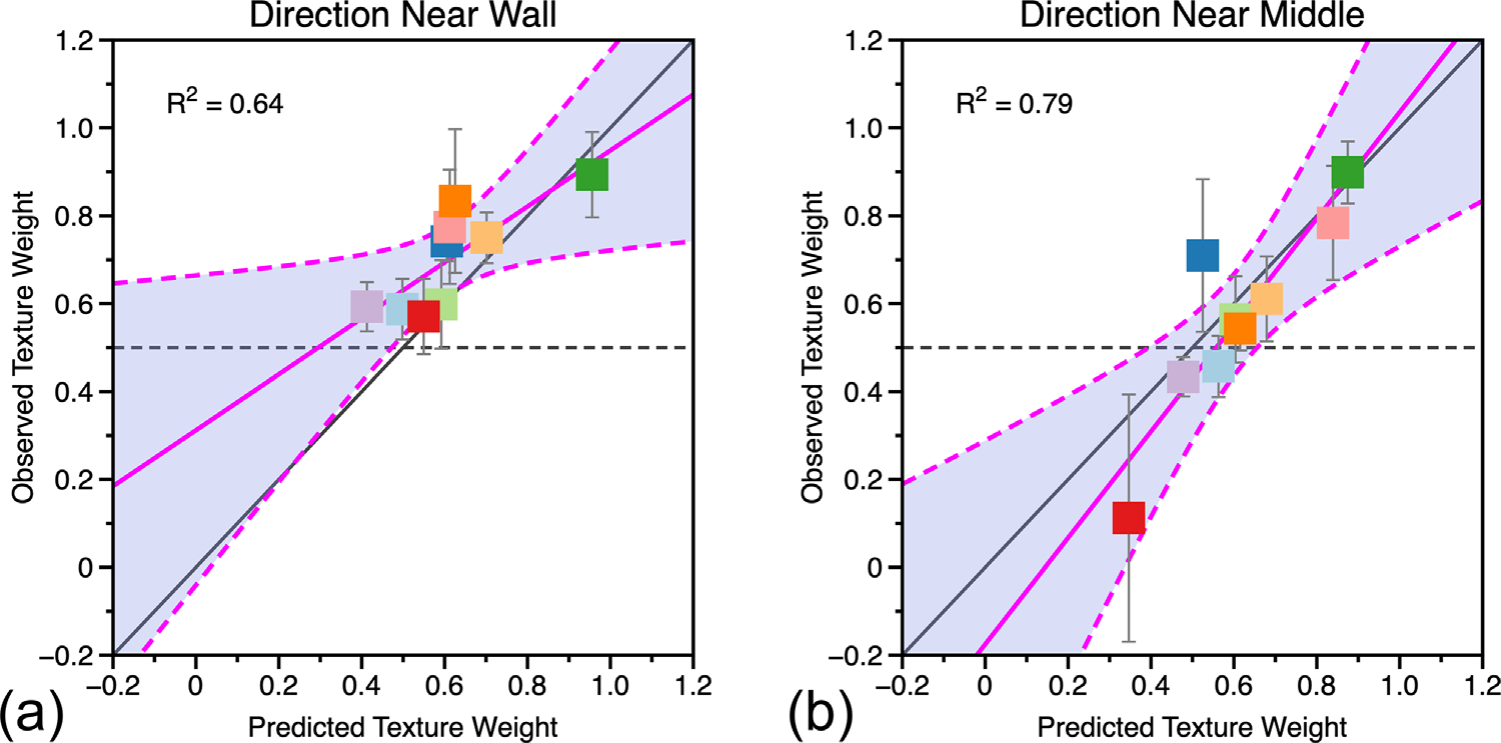
Predicted and observed texture weights for judgments of visual direction in the (a) “near wall” and (b) “near middle” conditions. Predicted weights are calculated from single cue sensitivities, observed weights from the data where both cues varied. If the combined cues data were perfectly predicted from the weighted averaging model, the data would fall on the solid black diagonal line in each plot. If the combined cue data were unrelated to single cue sensitivities, the data would fall (on average) along the dashed black line in each plot. The blue line in each plot shows a linear fit to the data with associated 95% confidence bounds (R^2^ value of the fit shown in text inset).

Figure 8 shows the predicted and observed texture weights for the ‘near wall’ and ‘near middle’ conditions using σ_*A*_ and σ_*B*_ derived from Equation 20. If the weighted averaging model perfectly predicted observers’ performance, all the data points should lie along the black diagonal line with a slope of one and intercept of zero. We fitted a linear model to the data by least squares. There was a significant linear relationship between predicted and observed texture cue weights for both the near wall, R^2^ = 0.64, F_(1, 7)_ = 12.4, *p* = 0.01, and near middle conditions, R^2^ = 0.79, F_(1, 7)_ = 26.0, *p* = 0.001.

### Perceived distance: Single cues

**Figure 9.**
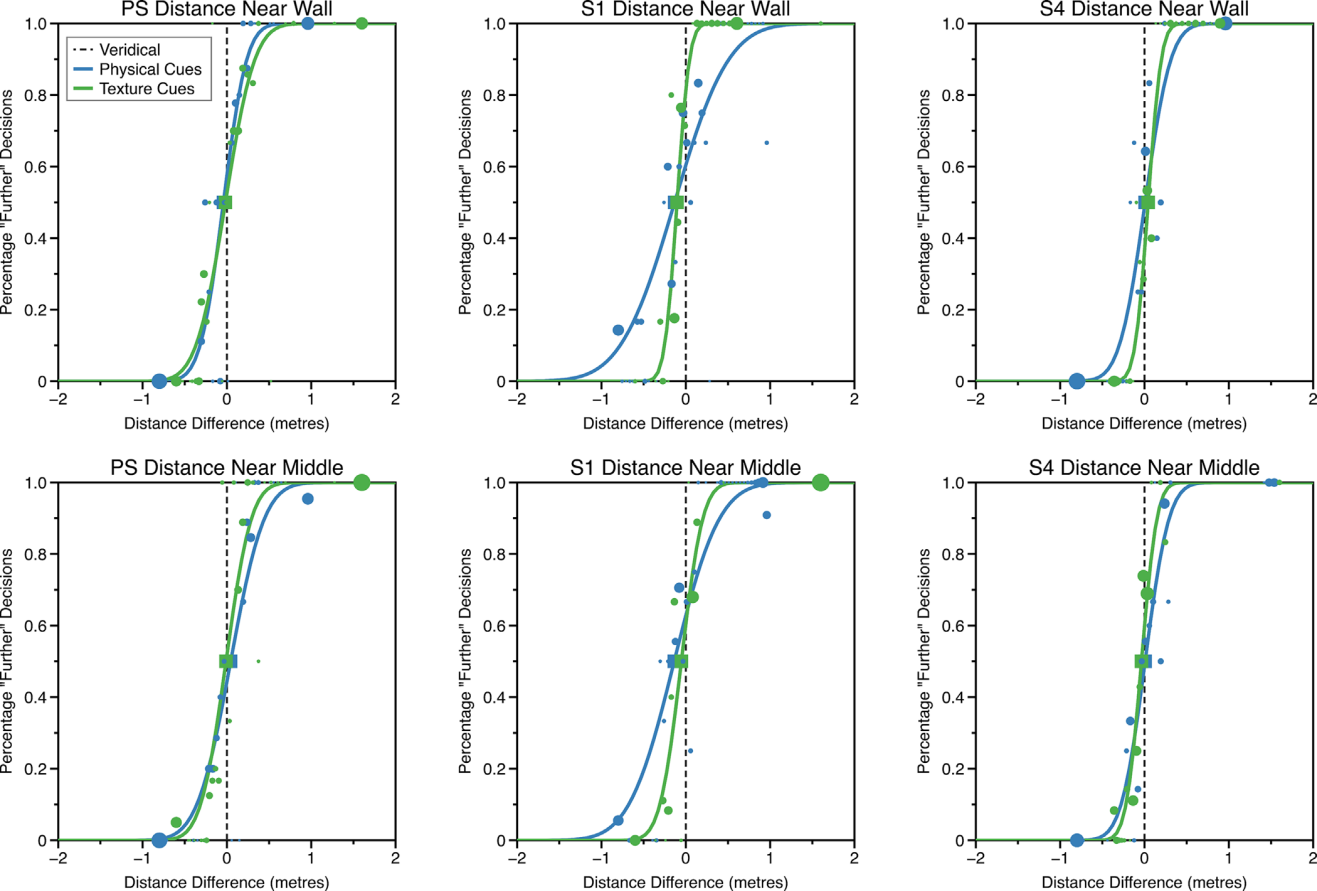
Example psychometric functions for physical and texture cues for judgments of distance (upper row “near wall” condition, lower row “near middle” condition). The circular marker area is proportional to the number of trials at that stimulus levels, as determined by the psi-marginal adaptive procedure. The squares represent the point of subjective equality (mean of the psychometric function). Error bars around the PSE show bootstrapped 95% confidence intervals (where these are not visible, they are smaller than the size of the marker). The vertical black dashed line shows “veridical” performance; the PSEs of the psychometric functions would fall on this line if the observer was unbiased in judging distance.

After converting changes in room scale factor between intervals 1 and 2 into differences in ball distance, from each class of cue, cumulative Gaussian functions were fitted in the same way as described elsewhere in this article. Example functions for three representative observers are shown in Figure 9. The observers are the same as in Figure 3, except for S2 who did not take part in the distance experiment. S2 has been replaced by S4, who is also an observer naïve to the purposes of the experiment, with little or no experience of psychophysical experiments.

### Perceived distance: Do observers optimally combine texture-based and physical-based cues?

**Figure 10.**
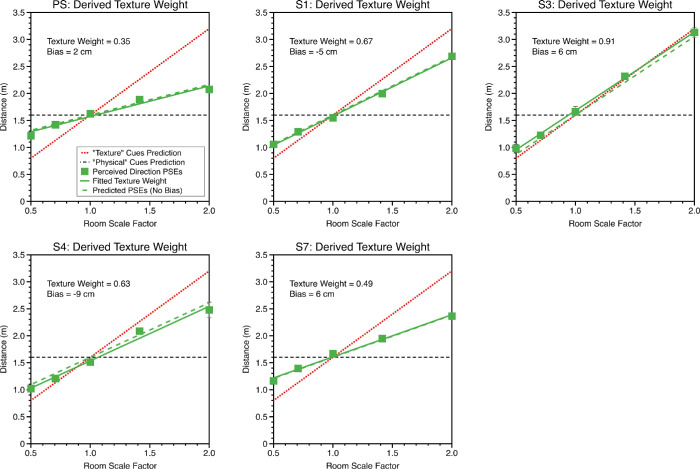
PSEs for perceived distance in the “near wall” condition when both cues varied are plotted against room scale factor along with a fit of equation 10 (green dots and line), which was used to determine relative cue weighting and level of perceptual bias (inset text). The horizontal dashed black line shows the prediction if an observer gave zero weight to texture-based cues, the dotted red line if the observer gave zero weight to physical cues (both assuming no perceptual bias). The dashed green line shows the predicted perceived distance if the observer weighted the cues as they did in the experiment but exhibited no perceptual bias.

**Figure 11.**
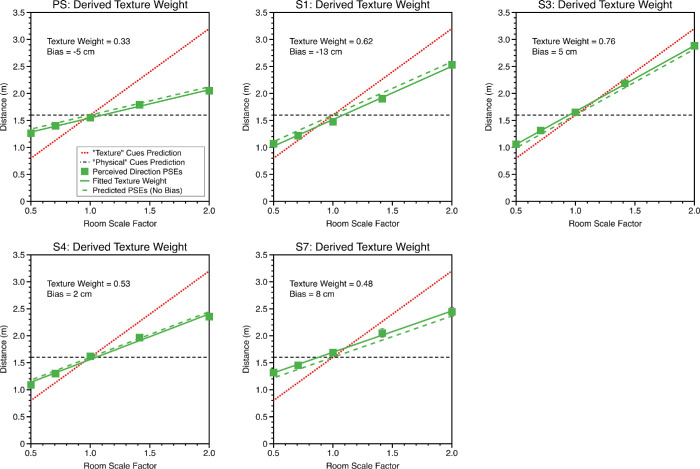
PSEs for perceived distance in the “near middle” condition using the same layout as for the “near wall” data in Figure 10.

Psychometric functions for perceived distance for each of the five room scale factors were fitted using the same procedure as described and a linear model fitting to estimate the cue weighting that participants used for their distance judgments when both cues varied (Equation 15). Figure 10 shows fits for the near wall condition and Figure 11 for the near middle condition. Consistent with the single cue threshold data, biases were universally small.

**Figure 12.**
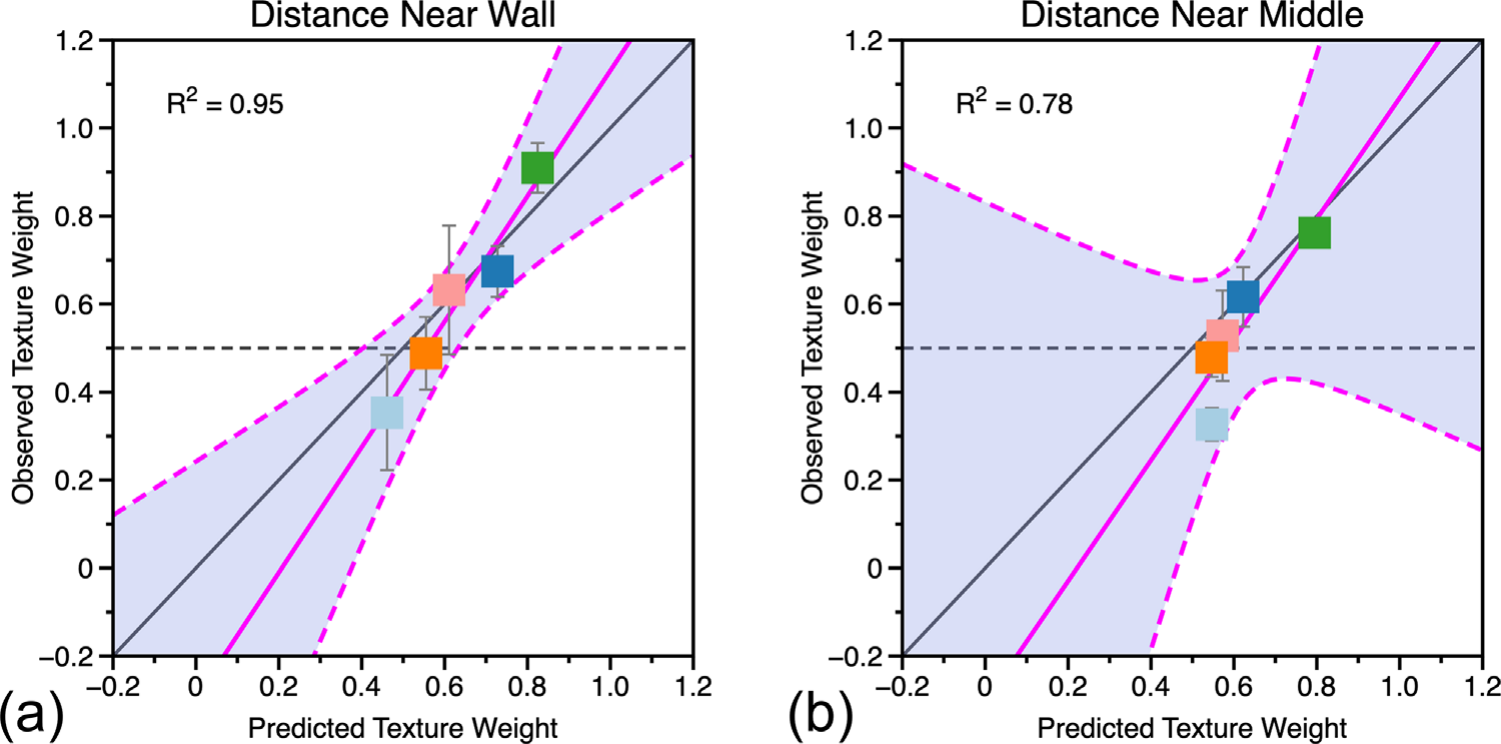
Predicted and observed texture weights for judgments of distance in the (a) “near wall” and (b) “near middle” conditions. Predicted weights are calculated from single cue sensitivities, observed weights from the data where both cues varied. If the combined cue data were perfectly predicted from the weighted averaging model, the data would fall on the solid black diagonal line in each plot. If the combined cue data were unrelated to single cue sensitivities, the data would fall along the dashed black line in each plot. The pink line in each plot shows a linear fit to the data with associated 95% confidence bounds (R^2^ value of the fit shown in text inset).

Figure 12 shows predicted and observed texture weights for the distance judgments made near the wall (left) and near the middle of the room (right). There was a significant linear relationship between predicted and observed texture cue weights for both the near wall, R^2^ = 0.95, F_(1, 3)_ = 56.6, *p* = 0.005, and near middle conditions, R^2^ = 0.78, F_(1, 3)_ = 10.9, *p* = 0.045.

### Are estimates of distance and direction consistent with one another?

To see whether observers’ judgments of direction and distance are mutually consistent, they need to be in common units (either distance or direction). Therefore, for the following we have converted observers’ distance judgments (and the standard deviation around these values) into units of visual angle. For each room scale factor this angle is calculated as the visual direction observers would perceive the ball to be in from view zone 2, if it were at the position defined by the PSE in the distance judgment (see Figure 2).

(21) $$\theta_{dist}=\mathrm{ta}n^{-1}\left(\frac{D_{PSE}}{D_{walked}}\right)$$

**Figure 13.**
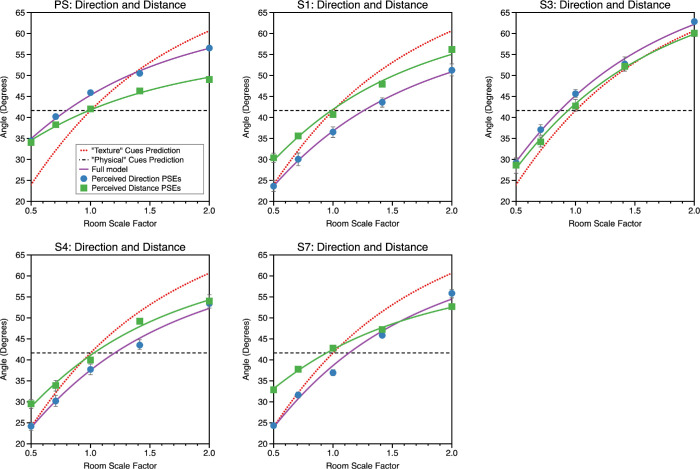
Data for the direction and distance estimation tasks, for the “near wall” condition, plotted in common units of visual angle. The solid lines represent a model fit in which the observers are modelled as exhibiting both differential cue weighting and differential bias for their estimates of both direction and distance. This was the model that gave the best fit overall across observers (see text). The red dotted line in each plot represents the prediction if physical cues had zero weighting and the dashed black line shows the case where texture cues had zero weighting: these predictions assume no bias in perceptual estimates.

**Figure 14.**
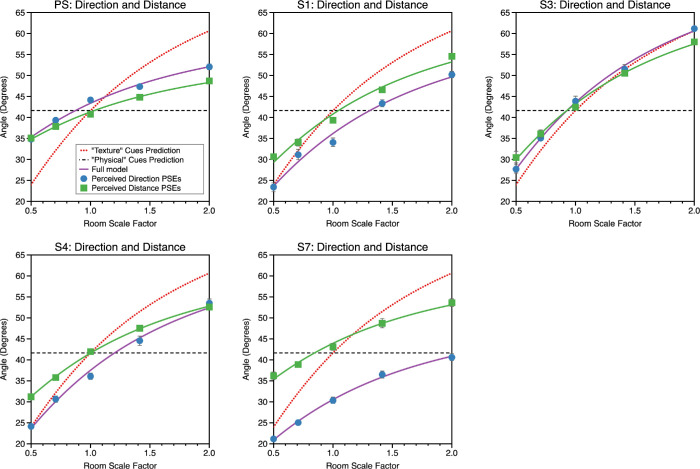
Data for the direction and distance estimation tasks, for the “near middle” condition. The layout is as for the “near wall” condition shown in Figure 13.

Here, *D_PSE_* is the observer's PSE for the distance judgment for a given scale factor while *D_walked_* is the physical distance from zone 1 to zone two, and θ_*dist*_ the visual direction the observer would see the ball in from view zone 2, if they were to judge *D_walked_* correctly. Data are plotted for the near wall condition in Figure 13 and the near middle condition in Figure 14. The error bars are asymmetric; this is because the conversion from distance to direction is a nonlinear transform.

In almost all instances in Figures 13 and 14, the perceived visual direction of an object is inconsistent with its perceived distance. In the Introduction, we discussed two ways in which estimates of distance and direction might be mutually inconsistent. First, physical and texture cues could be weighted differently for the two types of task, and second, estimates of distance and direction might be differentially biased. A model comparison approach was used to examine these possibilities, by fitting Equation 15 to the data by least squares using the *fminsearch* function in *Matlab* under four models. Model 1 allowed differential weighting and bias between distance and direction estimates (four free parameters). Model 2 allowed differential cue weighting but constrained the bias terms to be identical (three free parameters). Model 3 allowed differential cue bias but constrained the weighting terms to be identical (three free parameters). Model 4 constrained the weighting and bias terms to be identical (two free parameters).

The log-likelihood of each model was determined and the Akaike Information Criteria (AIC) and Bayesian Information Criteria (BIC) used to compare the relative likelihood of each model while taking into account the number of free parameters in each model. In this calculation, we have taken the mean of the asymmetric standard deviation for θ_*dist*_ in calculating the likelihoods (see Rosas et al., 2004 for an extended discussion). We present both the AIC (Table 1) and BIC (Table 2) values but the conclusions are the same. Lower numbers represent a high relative likelihood. As can be seen, regardless of the metric, model 1, with differential weighting and bias, provided the best account of the data. Model 1 was better than model 2 (constrained bias) in 90% of instances, it was better than model 3 (constrained weighting) in 80% of instances and was better than model 4, (constrained weighting and bias) in 100% of instances. Model 1 is shown as solid lines in Figures 13 and 14.

**Table 1.** Akaike information criteria (AIC) values for each candidate model for all observers in both room positions. *Notes*: *Model with the highest relative likelihood.

	Near wall				Near middle			
AIC observer	Model 1	Model 2	Model 3	Model 4	Model 1	Model 2	Model 3	Model 4
PS	34.26*	317.91	103.41	980.54	42.50*	105.26	49.70	416.41
S1	43.88	42.89*	72.16	316.68	158.79*	171.66	224.27	478.38
S3	26.01	25.34	24.31*	67.28	24.78*	74.75	51.33	84.48
S4	53.62*	65.20	86.75	135.59	27.58*	161.67	350.54	593.78
S7	49.61*	306.19	554.0	809.66	21.64*	27.23	437.86	Infinite

**Table 2.** Bayesian information criteria (BIC) values for each candidate model for all observers in both room positions. *Notes*: The infinite value is due to the model effectively having zero probability of producing the data for this observer. *Model with the highest relative likelihood.

	Near wall				Near middle			
BIC observer	Model 1	Model 2	Model 3	Model 4	Model 1	Model 2	Model 3	Model 4
PS	35.47*	318.81	104.32	981.05	43.71*	106.17	50.61	417.02
S1	45.1	43.8*	73.07	317.28	160.00*	172.57	225.18	478.98
S3	27.22	26.24	25.22*	67.88	25.99*	75.66	52.24	84.09
S4	54.83*	66.11	87.66	136.20	28.79*	162.58	351.45	594.39
S7	50.82*	307.09	554.9	820.26	22.87*	28.13	438.76	Infinite

## Discussion

Visual direction is, in some senses, the poor relation in 3D vision; there are many fewer studies examining the cues used to judge the visual direction of objects, and how visual direction is updated in the visual system, than there are for distance and depth. This neglect is understandable, given that vision has primarily been studied in static observers. With the use of virtual reality, it is now possible to parametrically manipulate the cues available to freely moving observers and examine how information is utilized to make perceptual judgments (Glennerster et al., 2006; Scarfe & Glennerster, 2015, 2019; Svarverud et al., 2012; Svarverud et al., 2010). Here, we have shown how estimates of the visual direction of a target can be influenced by two different classes of cue, namely, texture-based cues such as the distance of an object relative to the back wall of the room, and physical-based cues such as vergence and motion parallax scaled by proprioceptive information about the distance the observer moves. It is logical to separate these two cues and to examine their independent contribution to judgments of visual direction because they are quite different.

We also examined whether the estimates for distance and direction were consistent with one another. We found that they were not and that under the assumptions of the weighted averaging model this inconsistency could be attributed to two factors. First, cues were differentially weighted for the two types of judgments and, second, the judgments of visual direction exhibited perceptual bias, whereas judgements of distance did not. In the context of the weighted averaging model, differential cue weighting is relatively uncontroversial (Knill, 2005), whereas differential bias is the focus of active debate. This is because cues are typically assumed a priori to be unbiased (Landy et al., 1995; Maloney & Landy, 1989), with any bias being attributed to conflicting sources of sensory information or decision level bias (Watt et al., 2005). However, the perceptual bias exhibited in the present study is consistent with a large range of experimental data gathered with both real-world stimuli (Bradshaw et al., 2000; Cuijpers et al., 2000; Koenderink et al., 2002; Koenderink et al., 2000) and that collected with carefully controlled simulated stimuli (Watt et al., 2005).

**Figure 15.**
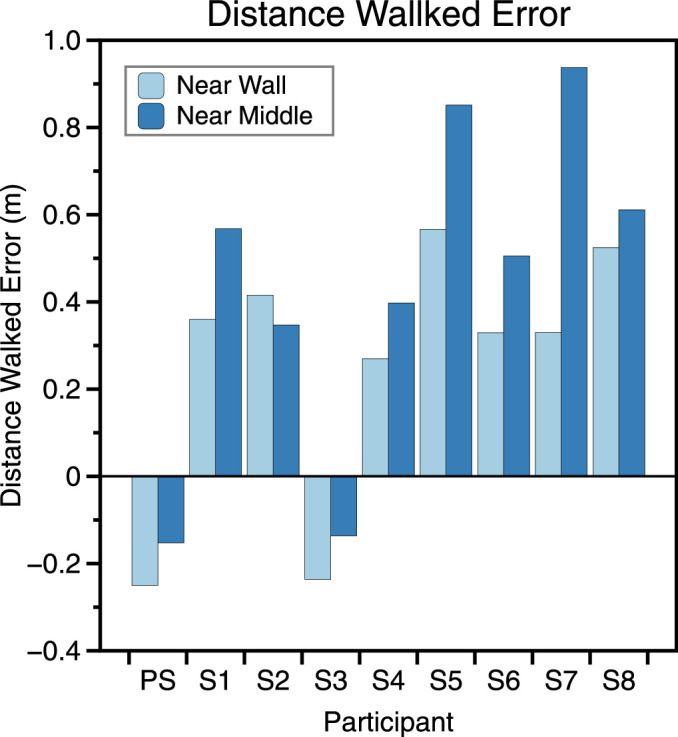
For each room position, this shows the extent of the misestimation in distance walked that would be required to account for each participant's misestimation of visual direction.

As a result, it is now accepted that bias needs to be accounted for in any explanation of how observers combine cues to make perceptual estimates (Ernst & Di Luca, 2011; Scarfe & Hibbard, 2011). The etiology of perceptual bias and how this might be incorporated into models of sensory cue combination is an area of active debate (Di Luca et al., 2010; Domini & Caudek, 2009; Domini et al., 2006; Saunders & Chen, 2015; Scarfe & Hibbard, 2011; Tassinari & Domini, 2008; Todd, 2015; Todd et al., 2010). In our experiment, the visual direction judgment contains a potential source of bias that does not exist in the distance judgment (indeed, for the distance judgment when the room does not expand or contract the cues in the first and second interval of a trial are so similar that it is difficult for participants’ judgments to be biased). This extra element is the estimate of distance walked, a parameter that has long been considered as a possible cause of biases in spatial updating tasks (Fukusima et al., 1997; Gogel, 1990; Gogel & Tietz, 1979). A parsimonious explanation of the biases is therefore that observers misestimated how far they walked across the room. Figure 15 plots the amount by which observers would have to misestimate distance walked to account for their estimates of visual direction when the room did not change scale between intervals 1 and 2. As can be seen, if all error is attributed to misestimating distance walked, most observers would have overestimated how far they walked across the room.

Our data show that it is possible to find good evidence (compatible with existing research) that observers are combining cues optimally consistent with weighted averaging, yet at the same time, good evidence against the hypothesis that the visual system builds a 3D reconstruction for different tasks. These results contradict the mantra that arose from early papers on cue combination, which assumed that the goal of the process was to yield a single interpretation of the scene, for example, a depth map (Landy et al., 1995). However, they are entirely consistent with the results of many studies which show that perceptual judgments depend on the task and that one should not expect consistency across tasks (Bradshaw et al., 2000; Glennerster et al., 1996; Koenderink et al., 2002; Smeets & Brenner, 2008). This is reflected in the wider Bayesian perspective in which optimality can depend on the perceptual judgments the observer is making (Schrater & Kersten, 2000).

With the predictions of an optimal cue combination model such as weighted averaging, it is essential to consider the criteria for a good fit. Often, the claim is made that the cue combination data fit well with the model, and the reader is left to judge the fit of the model to the data by eye (e.g., Ernst & Banks, 2002; Hillis et al., 2002; Hillis et al., 2004). Indeed, this has been recommended as a way in which to judge the degree of optimality in cue combination (Rohde et al., 2016). Counterexamples include Burge, Girshick and Banks (2010), who examined the perception of slant from disparity and haptic cues and reported an R^2^ of 0.60 for predicted versus observed sensitivity to slant (their Figure 3a); Knill and Saunders (2003), who examined the perception of slant from disparity and texture and reported R^2^ values between 0.15 and 0.46 for different base slants (their Figure 13); and Svarverud et al. (2010), who examined texture and physical cues to distance and reported R^2^ values of about 0.95 (their Figure 6), although the R^2^ values in Svarverud et al. (2010) would have to be adjusted in the light of Equation 20 before any firm conclusion can be made as the correction to cue reliabilities was not made in that paper.

Although the data from the present study fall squarely within the range of reported R^2^ values in the literature, we do not consider this good evidence that cues are combined optimally according to weighted averaging. First, the R^2^ does not provide a measure of how well the data fit the numerical predictions made by weighted averaging. For example, there could be a perfect linear relationship between predicted and observed cue weighting, in the sense that R^2^ = 1, but the observed data could be offset relative to the predicted data by an arbitrary constant value. Thus, with an R^2^ = 1, the predicted and observed data could provide wildly different absolute values. Additionally, there are numerous candidate models of how human observers might combine different sources of sensory information (Jones, 2016). As such, deciding whether a model fits well should be done with reference to alternatives (de Winkel et al., 2018; Lovell et al., 2012).

When this is done (and it is comparatively rare) it becomes clear how hard it is to distinguish certain models from one another due to their correlated predictions of combined cues performance (Arnold et al., 2019; Scarfe & Glennerster, 2018). Scarfe (2020) discusses this problem in much greater detail. Here, our aim was to determine whether one could find good evidence for observers combining cues optimally, according to the criteria of published research, for both judgments of direction and distance while at the same time assessing whether these judgments were mutually consistent with one another. We find that estimates can be judged as optimal, yet at the same time be mutually inconsistent.

## Supplementary Material

Supplement 1

## References

[bib1] Arnold, D. H., Petrie, K., Murray, C., & Johnston, A. (2019). Suboptimal human multisensory cue combination. *Scientific Reports**,* 9(1), 5155, 10.1038/s41598-018-37888-7. 30914673 PMC6435731

[bib2] Backus, B. T., & Matza-Brown, D. (2003). The contribution of vergence change to the measurement of relative disparity. *Journal of Vision**,* 3(11), 737–750, 10.1167/3.11.8. 14765957

[bib3] Bradshaw, M. F., Parton, A. D., & Glennerster, A. (2000). The task-dependent use of binocular disparity and motion parallax information. *Vision Research**,* 40(27), 3725–3734. 11090665 10.1016/s0042-6989(00)00214-5

[bib4] Brainard, D. H. (1997). The Psychophysics Toolbox. *Spatial Vision**,* 10(4), 433–436, https://www.ncbi.nlm.nih.gov/pubmed/9176952. 9176952

[bib5] Brenner, E., & van Damme, W. J. (1998). Judging distance from ocular convergence. *Vision Research**,* 38(4), 493–498, https://www.ncbi.nlm.nih.gov/pubmed/9536373. 9536373 10.1016/s0042-6989(97)00236-8

[bib6] Burge, J., Girshick, A. R., & Banks, M. S. (2010). Visual-haptic adaptation is determined by relative reliability. *Journal of Neuroscience**,* 30(22), 7714–7721, 10.1523/JNEUROSCI.6427-09.2010. 20519546 PMC3056491

[bib7] Cuijpers, R. H., Kappers, A. M., & Koenderink, J. J. (2000). Large systematic deviations in visual parallelism. *Perception**,* 29(12), 1467–1482, 10.1068/p3041. 11257970

[bib8] de Winkel, K. N., Katliar, M., Diers, D., & Bulthoff, H. H. (2018). Causal inference in the perception of verticality. *Scientific Reports**,* 8(1), 5483, 10.1038/s41598-018-23838-w. 29615728 PMC5882842

[bib9] Di Luca, M., Domini, F., & Caudek, C. (2010). Inconsistency of perceived 3D shape. *Vision Research**,* 50(16), 1519–1531, 10.1016/j.visres.2010.05.006. 20470815

[bib10] Domini, F., & Caudek, C. (2009). The intrinsic constraint model and Fechnerian sensory scaling. *Journal of Vision**,* 9(2), 25, 21–15, 10.1167/9.2.25. 19271935

[bib11] Domini, F., Caudek, C., & Tassinari, H. (2006). Stereo and motion information are not independently processed by the visual system. *Vision Research**,* 46(11), 1707–1723, 10.1016/j.visres.2005.11.018. 16412492

[bib12] Ernst, M. O., & Banks, M. S. (2002). Humans integrate visual and haptic information in a statistically optimal fashion. *Nature**,* 415(6870), 429–433, 10.1038/415429a. 11807554

[bib13] Ernst, M. O., & Bulthoff, H. H. (2004). Merging the senses into a robust percept. *Trends in Cognitive Science**,* 8(4), 162–169, 10.1016/j.tics.2004.02.002. 15050512

[bib14] Ernst, M. O., & Di Luca, M. (2011). Multisensory perception: From integration to remapping. In: J. Trommershauser, K. P. Körding, & M. S. Landy (Eds.), *Sensory cue integration* (pp. 224–250). Oxford, UK: Oxford University Press.

[bib15] Foo, P., Warren, W. H., Duchon, A., & Tarr, M. J. (2005). Do humans integrate routes into a cognitive map? Map- versus landmark-based navigation of novel shortcuts. *Journal of Experimental Psychology. Learning, Memory, and Cognition**,* 31(2), 195–215, 10.1037/0278-7393.31.2.195. 15755239

[bib16] Frissen, I., Campos, J. L., Souman, J. L., & Ernst, M. O. (2011). Integration of vestibular and proprioceptive signals for spatial updating. *Experimental Brain Research**,* 212(2), 163–176, 10.1007/s00221-011-2717-9. 21590262

[bib17] Fukusima, S. S., Loomis, J. M., & DaSilva, J. A. (1997). Visual perception of egocentric distance as assessed by triangulation. *Journal of Experimental Psychology. Human Perception and Performance**,* 23(1), 86–100. 9090148 10.1037//0096-1523.23.1.86

[bib18] Gilson, S. J., Fitzgibbon, A. W., & Glennerster, A. (2011). An automated calibration method for non-see-through head mounted displays. *J Neuroscience Methods**,* 199(2), 328–335, 10.1016/j.jneumeth.2011.05.011. PMC3142613 21620891

[bib19] Giudice, N. A., Betty, M. R., & Loomis, J. M. (2011). Functional equivalence of spatial images from touch and vision: Evidence from spatial updating in blind and sighted individuals. *Journal of Experimental Psychology. Learning, Memory, and Cognition**,* 37(3), 621–634, 10.1037/a0022331. PMC5507195 21299331

[bib20] Glennerster, A., Rogers, B. J., & Bradshaw, M. F. (1996). Stereoscopic depth constancy depends on the subject's task. *Vision Research**,* 36(21), 3441–3456, https://www.ncbi.nlm.nih.gov/pubmed/8977011. 8977011 10.1016/0042-6989(96)00090-9

[bib21] Glennerster, A., Tcheang, L., Gilson, S. J., Fitzgibbon, A. W., & Parker, A. J. (2006). Humans ignore motion and stereo cues in favor of a fictional stable world. *Current Biology**,* 16(4), 428–432, 10.1016/j.cub.2006.01.019. 16488879 PMC2833396

[bib22] Gogel, W. C. (1990). A theory of phenomenal geometry and its applications. *Perception Psychophysics**,* 48(2), 105–123, 10.3758/bf03207077. 2385484

[bib23] Gogel, W. C., & Tietz, J. D. (1979). A comparison of oculomotor and motion parallax cues of egocentric distance. *Vision Research**,* 19(10), 1161–1170, 10.1016/0042-6989(79)90013-0. 550575

[bib24] Harris, J. M. (2004). Binocular vision: Moving closer to reality. *Philosophical Transactions. Services A, Mathematical, Physical, and Engineering Science**,* 362(1825), 2721–2739, 10.1098/rsta.2004.1464. 15539367

[bib25] Hartley, R., & Zisserman, A. (2000). *Multiple view geometry in computer vision*. Cambridge, UK: Cambridge University Press.

[bib26] Hillis, J. M., Ernst, M. O., Banks, M. S., & Landy, M. S. (2002). Combining sensory information: Mandatory fusion within, but not between, senses. *Science**,* 298(5598), 1627–1630. 12446912 10.1126/science.1075396

[bib27] Hillis, J. M., Watt, S. J., Landy, M. S., & Banks, M. S. (2004). Slant from texture and disparity cues: Optimal cue combination. *Journal of Vision**,* 4(12), 967–992, 10.1167/4.12.1. 15669906

[bib28] Howard, I. P. (2008). Vergence modulation as a cue to movement in depth. *Spatial Vision**,* 21(6), 581–592, 10.1163/156856808786451417. 19017484

[bib29] Howard, I. P., & Rogers, B. J. (2002). *Seeing in depth: Depth perception* (Vol. 2). I Porteous: University of Toronto Press.

[bib30] Jones, P. R. (2016). A tutorial on cue combination and Signal Detection Theory: Using changes in sensitivity to evaluate how observers integrate sensory information. *Journal of Mathematical Psychology**,* 73, 117–139.

[bib31] Klatzky, R. L., Lippa, Y., Loomis, J. M., & Golledge, R. G. (2003). Encoding, learning, and spatial updating of multiple object locations specified by 3-D sound, spatial language, and vision. *Experimental Brain Research**,* 149(1), 48–61, 10.1007/s00221-002-1334-z. 12592503

[bib32] Klatzky, R. L., Loomis, J. M., Beall, A. C., Chance, S. S., & Golledge, R. G. (1998). Spatial updating of self-position and orientation during real, imagined, and virtual locomotion. *Psychological Science**,* 9(4), 293–298, 10.1111/1467-9280.00058.

[bib33] Kleiner, M., Brainard, D., & Pelli, D. (2007). What's new in Psychtoolbox-3? [Meeting Abstract]. *Perception**,* 36, 14.

[bib34] Klier, E. M., Hess, B. J., & Angelaki, D. E. (2008). Human visuospatial updating after passive translations in three-dimensional space. *Journal of Neurophysiology**,* 99(4), 1799–1809, 10.1152/jn.01091.2007. 18256164 PMC3835451

[bib35] Knill, D. C. (2005). Reaching for visual cues to depth: The brain combines depth cues differently for motor control and perception. *Journal of Vision**,* 5(2), 103–115, 10.1167/5.2.2. 15831071

[bib36] Knill, D. C., & Saunders, J. A. (2003). Do humans optimally integrate stereo and texture information for judgments of surface slant? *Vision Research**,* 43(24), 2539–2558. 13129541 10.1016/s0042-6989(03)00458-9

[bib37] Koenderink, J. J., van Doorn, A. J., Kappers, A. M., & Lappin, J. S. (2002). Large-scale visual frontoparallels under full-cue conditions. *Perception**,* 31(12), 1467–1475, 10.1068/p3295. 12916671

[bib38] Koenderink, J. J., van Doorn, A. J., & Lappin, J. S. (2000). Direct measurement of the curvature of visual space. *Perception**,* 29(1), 69–79, 10.1068/p2921. 10820592

[bib39] Kontsevich, L. L., & Tyler, C. W. (1999). Bayesian adaptive estimation of psychometric slope and threshold. *Vision Research**,* 39(16), 2729–2737, 10.1016/S0042-6989(98)00285-5. 10492833

[bib40] Landy, M. S., Maloney, L. T., Johnston, E. B., & Young, M. (1995). Measurement and modeling of depth cue combination: In defense of weak fusion. *Vision Research**,* 35(3), 389–412, https://www.ncbi.nlm.nih.gov/pubmed/7892735. 7892735 10.1016/0042-6989(94)00176-m

[bib41] Loomis, J. M., Da Silva, J. A., Fujita, N., & Fukusima, S. S. (1992). Visual space perception and visually directed action. *J Exp Psychol Hum Percept Perform**,* 18(4), 906–921, https://www.ncbi.nlm.nih.gov/pubmed/1431754. 1431754 10.1037//0096-1523.18.4.906

[bib42] Loomis, J. M., Klatzky, R. L., Avraamides, M., Lippa, Y., & Golledge, R. G. (2007). Functional equivalence of spatial images produced by perception and spatial language. In: *Spatial processing in navigation, imagery and perception*. New York: Springer.

[bib43] Loomis, J. M., Klatzky, R. L., Philbeck, J. W., & Golledge, R. G. (1998). Assessing auditory distance perception using perceptually directed action. *Perception Psychophysics**,* 60(6), 966–980, https://www.ncbi.nlm.nih.gov/pubmed/9718956. 9718956 10.3758/bf03211932

[bib44] Loomis, J. M., & Philbeck, J. W. (2008). Measuring spatial perception with spatial updating and action. In M. Behrmann, R. L. Klatzky, & B. Macwhinney (Eds.), *Embodiment, ego-space, and action* (pp. 1–43). Hove, UK: Psychology Press.

[bib45] Lovell, P. G., Bloj, M., & Harris, J. M. (2012). Optimal integration of shading and binocular disparity for depth perception. *Journal of Vision**,* 12(1), 1, 10.1167/12.1.1. 22214563

[bib46] Maloney, L. T., & Landy, M. S. (1989). A statistical framework for robust fusion of depth information. Proceedings of SPIE 1199, Visual Communications and Image Processing IV, Philadelphia, PA.

[bib47] Mayne, R. (1974). A systems concept of the vestibular organs. In H. H. Kornhuber (Ed.), *Handbook of sensory physiology* (Vol. 2, pp. 493–580). New York: Springer.

[bib48] Medendorp, W. P. (2011). Spatial constancy mechanisms in motor control. *Philosophical Transactions of the Royal Society of London. Series B, Biological Sciences**,* 366(1564), 476–491, 10.1098/rstb.2010.0089. 21242137 PMC3030827

[bib49] Mon-Williams, M., Tresilian, J. R., & Roberts, A. (2000). Vergence provides veridical depth perception from horizontal retinal image disparities. *Experimental Brain Research**,* 133(3), 407–413, https://www.ncbi.nlm.nih.gov/pubmed/10958531. 10958531 10.1007/s002210000410

[bib50] Murphy, A. P., Ban, H., & Welchman, A. E. (2013). Integration of texture and disparity cues to surface slant in dorsal visual cortex. *Journal of Neurophysiology**,* 110(1), 190–203, 10.1152/jn.01055.2012. 23576705 PMC3727040

[bib51] Nardini, M., Jones, P., Bedford, R., & Braddick, O. (2008). Development of cue integration in human navigation. *Current Biology**,* 18(9), 689–693, 10.1016/j.cub.2008.04.021. 18450447

[bib52] Pelli, D. G. (1997). The VideoToolbox software for visual psychophysics: Transforming numbers into movies. *Spatial Vision**,* 10(4), 437–442, 9176953

[bib53] Prins, N. (2013). The psi-marginal adaptive method: How to give nuisance parameters the attention they deserve (no more, no less). *Journal of Vision**,* 13(7), 3, 10.1167/13.7.3. 23750016

[bib54] Prins, N., & Kingdom, F. A. A. (2009). *Palamedes**:* *Matlab* *routines for analyzing psychophysical data.* Available: www.palamedestoolbox.org.

[bib55] Rauschecker, A. M., Solomon, S. G., & Glennerster, A. (2006). Stereo and motion parallax cues in human 3D vision: Can they vanish without a trace? *Journal of Vision**,* 6(12), 1471–1485, 10.1167/6.12.12. 17209749

[bib56] Richards, W., & Miller, J. F. (1969). Convergence as a cue to depth. *Perception & Psychophysics**,* 5(5), 317–20.

[bib57] Rieser, J. J., & Rider, E. (1991). Young children's spatial orientation with respect to multiple targets when walking without vision. *Developmental Psychology**,* 27, 97–107.

[bib58] Rohde, M., van Dam, L. C. J., & Ernst, M. (2016). Statistically optimal multisensory cue integration: A practical tutorial. *Multisensory Research**,* 29(4–5), 279–317, https://www.ncbi.nlm.nih.gov/pubmed/29384605. 29384605 10.1163/22134808-00002510

[bib59] Rosas, P., Wichmann, F. A., & Wagemans, J. (2004). Some observations on the effects of slant and texture type on slant-from-texture. *Vision Research**,* 44(13), 1511–1535, 10.1016/j.visres.2004.01.013. 15126062

[bib60] Saunders, J. A., & Chen, Z. (2015). Perceptual biases and cue weighting in perception of 3D slant from texture and stereo information. *Journal of Vision**,* 15(2), 14, 10.1167/15.2.14. 25761332

[bib61] Scarfe, P. (2020). Experimentally disambiguating models of sensory cue integration. *bioRxiv*, 2020.09.01.277400 10.1101/2020.09.01.277400. PMC8762719 35019955

[bib62] Scarfe, P., & Glennerster, A. (2015). Using high-fidelity virtual reality to study perception in freely moving observers. *Journal of Vision**,* 15(9), 3, 10.1167/15.9.3. 26161632

[bib63] Scarfe, P., & Glennerster, A. (2018). Experimentally disambiguating models of sensory cue combination. *Journal of Vision**,* 18(10), 788–788. 10.1167/jov.22.1.5 PMC8762719 35019955

[bib64] Scarfe, P., & Glennerster, A. (2019). The science behind virtual reality displays. *Annual Review of Vision Science**,* 5, 529–547, 10.1146/annurev-vision-091718-014942. 31283449

[bib65] Scarfe, P., & Hibbard, P. B. (2011). Statistically optimal integration of biased sensory estimates. *Journal of Vision**,* 11(7), 12, 10.1167/11.7.12. 21670095

[bib66] Schrater, P. R., & Kersten, D. (2000). How optimal depth cue integration depends on the task. *International Journal of Computer Vision**,* 40(1), 73–91.

[bib67] Shapiro, L. S., Zisserman, A., & Brady, M. (1995). 3d motion recovery via affine epipolar geometry. *International Journal of Computer Vision**,* 16(2), 147–182, 10.1007/Bf01539553.

[bib68] Siegle, J. H., Campos, J. L., Mohler, B. J., Loomis, J. M., & Bulthoff, H. H. (2009). Measurement of instantaneous perceived self-motion using continuous pointing. *Experimental Brain Research**,* 195(3), 429–444, 10.1007/s00221-009-1805-6. 19396591

[bib69] Smeets, J. B., & Brenner, E. (2008). Why we don't mind to be inconsistent. In P. Cavo & T. Gomila (Eds.), *Handbook of cognitive science - An embodied approach* (pp. 207–217). New York: Elsevier.

[bib70] Smeets, J. B., van den Dobbelsteen, J. J., de Grave, D. D., van Beers, R. J., & Brenner, E. (2006). Sensory integration does not lead to sensory calibration [Comparative Study Research Support, Non-U.S. Gov't]. *Proceedings of the National Academy of Sciences of the United States of America**,* 103(49), 18781–18786, 10.1073/pnas.0607687103. 17130453 PMC1693739

[bib71] Svarverud, E., Gilson, S., & Glennerster, A. (2012). A demonstration of ‘broken’ visual space. *PloS One**,* 7(3), e33782, 10.1371/journal.pone.0033782. 22479441 PMC3315588

[bib72] Svarverud, E., Gilson, S. J., & Glennerster, A. (2010). Cue combination for 3D location judgements. *Journal of Vision**,* 10(1), 5, 1–13, 10.1167/10.1.5. PMC2836116 20143898

[bib73] Swenson, H. A. (1932). The relative influence of accommodation and convergence in the judgment of distance. *Journal of General Psychology**,* 7, 360–380.

[bib74] Tassinari, H., & Domini, F. (2008). The intrinsic constraint model for stereo-motion integration. *Perception**,* 37(1), 79–95, 10.1068/p5501. 18399249

[bib75] Tcheang, L., Gilson, S. J., & Glennerster, A. (2005). Systematic distortions of perceptual stability investigated using immersive virtual reality. *Vision Research**,* 45(16), 2177–2189. 15845248 10.1016/j.visres.2005.02.006 PMC2833395

[bib76] Thompson, W. B., Willemsen, P., Gooch, A. A., Creem-Regehr, S. H., Loomis, J. M., & Beall, A. C. (2004). Does the quality of the computer graphics matter when judging distances in visually immersive environments. *Presence-Teleoperators and Virtual Environments**,* 13, 560–571.

[bib77] Todd, J. T. (2015). Can a Bayesian analysis account for systematic errors in judgments of 3D shape from texture? A reply to Saunders and Chen. *Journal of Vision**,* 15(9), 22, 10.1167/15.9.22. 26230984

[bib78] Todd, J. T., Christensen, J. C., & Guckes, K. M. (2010). Are discrimination thresholds a valid measure of variance for judgments of slant from texture? *Journal of Vision**,* 10(2), 20 21–18, 10.1167/10.2.20. 20462321

[bib79] Tresilian, J. R., & Mon-Williams, M. (2000). Getting the measure of vergence weight in nearness perception. *Experimental Brain Research**,* 132(3), 362–368, 10.1007/s002210000333. 10883384

[bib80] Tresilian, J. R., Mon-Williams, M., & Kelly, B. M. (1999). Increasing confidence in vergence as a cue to distance. *Proceedings of the Royal Society of London Series B, Biological Sciences**,* 266(1414), 39–44. 10081157 10.1098/rspb.1999.0601 PMC1689642

[bib81] Wallach, H., Stanton, L., & Becker, D. (1974). The compensation for movement-produced changes of object orientation. *Perception and Psychophysics**,* 15, 339–343.

[bib82] Watt, S. J., Akeley, K., Ernst, M. O., & Banks, M. S. (2005). Focus cues affect perceived depth. *Journal of Vision**,* 5(10), 834–862, 10.1167/5.10.7. 16441189 PMC2667386

[bib83] Wexler, M., Panerai, F., Lamouret, I., & Droulez, J. (2001). Self-motion and the perception of stationary objects. *Nature**,* 409(6816), 85–88, 10.1038/35051081. 11343118

[bib84] Wichmann, F. A., & Hill, N. J. (2001a). The psychometric function: I. Fitting, sampling, and goodness of fit. *Perception Psychophysics**,* 63(8), 1293–1313, https://www.ncbi.nlm.nih.gov/pubmed/11800458. 11800458 10.3758/bf03194544

[bib85] Wichmann, F. A., & Hill, N. J. (2001b). The psychometric function: II. Bootstrap-based confidence intervals and sampling. *Perception Psychophysics**,* 63(8), 1314–1329, https://www.ncbi.nlm.nih.gov/pubmed/11800459. 11800459 10.3758/bf03194545

